# Organism-wide cellular dynamics and epigenomic remodeling in mammalian aging

**DOI:** 10.1101/2025.05.12.653376

**Published:** 2025-05-15

**Authors:** Ziyu Lu, Zehao Zhang, Zihan Xu, Abdulraouf Abdulraouf, Wei Zhou, Junyue Cao

**Affiliations:** 1 Laboratory of Single Cell Genomics and Population Dynamics, The Rockefeller University, New York, NY, USA; 2 The David Rockefeller Graduate Program in Bioscience, The Rockefeller University, New York, NY, USA; 3 The Tri-Institutional M.D-Ph.D Program, New York, NY, USA; 4 Senior author; 5 Lead Contact

## Abstract

Aging leads to functional decline across tissues, often accompanied by profound changes in cellular composition and cell-intrinsic molecular states. However, a comprehensive catalog of how the population of individual cell types change with age and the associated epigenomic dynamics is lacking. Here, we constructed a single-cell chromatin accessibility atlas consisting of ~7 million cells from 21 tissue types spanning three age groups in both sexes. This dataset revealed 536 main cell types and 1,828 finer-grained subtypes, defined by unique chromatin accessibility landscapes at ~1.3 million cis-regulatory elements. We observed widespread remodeling of immune lineages, with increases in plasma cells and macrophages, and depletion of T and B cell progenitors. Additionally, non-immune cell populations, including kidney podocytes, ovary granulosa cells, muscle tenocytes and lung aerocytes, showed marked reductions with age. Meanwhile, many subtypes changed synchronously across multiple organs, underscoring the potential influence of systemic inflammatory signals or hormonal cues. At the molecular level, aging was marked by thousands of differentially accessible regions, with the most concordant changes shared across cell types linked to genes related to inflammation or development. Putative upstream factors, such as intrinsic shifts in transcription factor usages and extrinsic cytokine signatures, were identified. Notably, around 40% of aging-associated main cell types and subtypes showed sex-dependent differences, with tens of thousands of chromatin accessibility peaks altered exclusively in one sex. Together, these findings present a comprehensive framework of how aging reshapes the chromatin landscape and cellular composition across diverse tissues, offering a comprehensive resource for understanding the molecular and cellular programs underlying aging and supporting the exploration of targeted therapeutic strategies to address age-related dysfunction.

## Introduction:

Aging is the leading risk factor for many diseases, including neurodegenerative disorders, cardiovascular diseases, metabolic conditions, and cancer(1). This association underscores the potential of therapies targeting the aging process itself to delay or prevent various age-related diseases, rather than focusing on specific diseases or organs. However, this approach is challenging due to the complexity of aging, which involves widespread changes across various systems and organs (2, 3). Each organ consists of hundreds of unique cellular states with specialized functions, each undergoing varied aging-associated changes. Therefore, a systematic characterization of aging-associated cell states—including their genetic and epigenetic alterations underlying population dynamics—is essential for identifying cellular and molecular targets that could guide the development of targeted therapeutic interventions.

Recent advancements in single-cell genomics have significantly enhanced our ability to map aging-associated cell states. For example, single-cell transcriptomics analyses have enabled us to profile alterations in thousands of genes across millions of cells as they age (4–7). However, these analyses typically focus on protein-coding genes and overlook the impact of non-coding genetic elements in regulating cell-type-specific dynamics in aging. Moreover, current single-cell atlas studies often focus on a limited set of organs (*e.g.,* liver, kidney), largely because some other tissues, like the pancreas, are difficult to profile due to high RNA degradation (8, 9). This limitation impedes our ability to assess aging across the entire organism. As a result, we still lack a comprehensive analysis of cell population dynamics and chromatin landscape changes throughout the mammalian body for understanding how non-coding regions, particularly cis-regulatory DNA elements, affect cell states and population dynamics as they age.

Single-cell ATAC-seq(10), which analyzes genome-wide chromatin accessibility at the single-cell level, has enabled the mapping of the cell-type-specific chromatin landscape in a range of mammalian tissues(11–14), aiding in the identification of disease-associated non-coding regions and their corresponding cell types. Among varied scATAC-seq approaches, single-cell ATAC-seq by combinatorial indexing (*sci-ATAC-seq*), allows for high-throughput analysis of tens of thousands of cells from highly heterogeneous tissues in a single experiment(11). With a further optimized version of sci-ATAC-seq (EasySci-ATAC), we recently mapped the cell-type-specific chromatin landscape and aging-associated cell population changes in the mammalian brain(15). Building on this work, we now extend our analysis beyond a single organ to explore aging-associated changes in cell populations across the entire organism, identifying changes in cellular chromatin states, population dynamics, and associated genetic elements—including cis-regulatory elements and transcription factor motifs—across different ages and sexes.

## Results

### An organismal, single-cell chromatin accessibility atlas of aging

**Figure 1.**
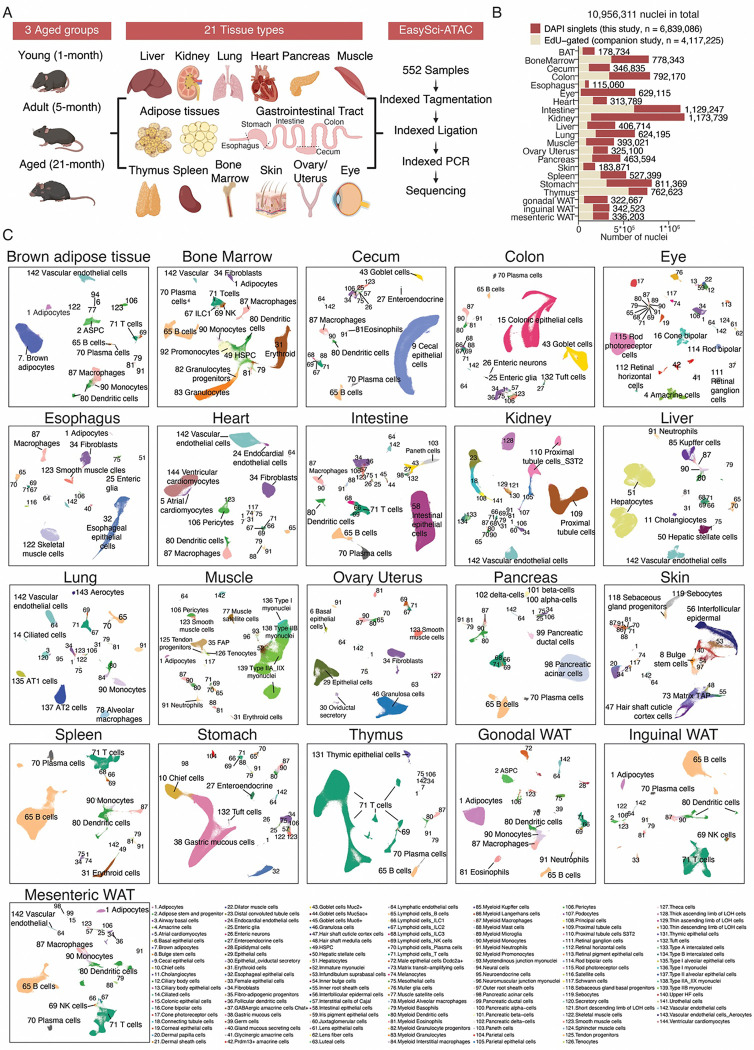
An organismal, single-cell chromatin accessibility atlas of aging. **(A)** Experimental scheme to construct an organismal cell atlas of chromatin accessibility across different ages and both sexes. **(B)** Barplot showing the number of cells profiled in each tissue, colored by the sources. DAPI singlets represent the global cell population investigated in this study. **(C)** UMAP visualization of cells from each tissue, colored by 144 unique cell types collapsed from all tissues.

To map aging-associated cell population dynamics and the cell-type-specific chromatin landscape changes at the organismal scale, we developed an optimized *EasySci-ATAC(15)* protocol that enables high-throughput analysis of mouse tissues across the entire organism (**Methods**). Next we applied this approach to profile 552 samples collected from thirty-two individual mice representing three age groups (1-month, 5-month, and 21-month)(Table S1). Each group contained 8–12 sex-balanced biological replicates, except for the female ovary and uterus samples. Twenty-one different tissue types spanning major biological systems were profiled, including the immune system (*i.e.,* bone marrow, spleen, thymus), the cardiovascular system (*i.e.,* heart), the respiratory system (*i.e.,* lung), the digestive system (*i.e.,* liver, pancreas, esophagus, stomach, intestine, cecum, colon), the urinary system (*i.e.,* kidney), the sensory system (*i.e.,* eye), the reproductive system (*i.e.,* ovary/uterus), the musculoskeletal system (*i.e.,* muscle), the integumentary system (*i.e.,* skin), and the adipose tissues, which includes brown adipose tissue (BAT) and white adipose tissues (WAT) in various anatomical locations (*i.e.,* gonadal, inguinal, and mesenteric) (Fig. 1A). Of note, the brain was not included here, as it has been extensively analyzed in our previous study(15). Also, to enable the investigation of newborn cell dynamics in a companion study, we labeled mice with 5-Ethynyl-2-deoxyuridine (EdU) before tissue collection, similar to our previous work (16). This allowed single-cell chromatin accessibility profiling of both sorted DAPI singlets (‘all’ cells, this study) and EdU+ nuclei (‘newborn’ cells, analyzed in the companion study) from the same set of samples.

The extracted nuclei from each tissue sample were sorted and combinatorial barcoded through indexed tagmentation, indexed ligation, and indexed PCR (Fig. 1A). The resulting libraries were sequenced on the Illumina NovaSeq 6000 system, yielding a total of 95 billion paired-end raw reads. After filtering out low-quality cells and doublets similar to our previous study (15) (Fig. S1A), we obtained chromatin accessibility profiles for a total of 10,956,311 single cells, including 6,839,086 DAPI singlets and 4,117,225 cells gated on EdU positivity (Fig. 1B and Fig. S1B). The median number of cells recovered per tissue range from 115,060 in the esophagus to 1,173,739 in the kidney (Fig. 1B). With a low sequencing duplication rate of only 20.1%, we detected a median of 3,031 unique ATAC-seq fragments per nucleus. On average, 29.8% of reads mapped to promoter regions (within ±1 kb of the transcription start site, TSS), aligning with performances from previous single-cell ATAC-seq studies (11, 15) (Fig. S1C and S1D). Also, the pseudo bulk ATAC-seq profiles are clustered by tissue type rather than by individual mice, indicating the low technical batch effect across individuals (Fig. S1E).

Next, we identified and annotated heterogeneous cell types within each tissue. Applying *SnapATAC2(17)*, we performed dimensionality reduction and Leiden clustering, and manually curated the annotation based on the gene accessibility of known markers (Table S2, Fig. S2). Of note, we utilized the full dataset containing 10,956,311 cells for clustering and annotations. Chromatin accessibility alone enabled the identification of diverse cell types, including tissues that are challenging to profile by scRNA-seq due to high RNase levels, such as ductal cells (marked by *Ccn2*), beta-cells (marked by *Gcg*), alpha-cells (marked by *Isn1*) and delta-cells (marked by *Sst*) in the pancreas (18, 19) (Fig. S2A). In total, we annotated 536 organ-level main cell types, which were consolidated into 144 unique categories, with a median of 25 cell types per tissue and 4,860 nuclei per cell type (Fig. 1C). While most cell types were restricted to one organ (n = 102), we identified eighteen broadly distributed cell types shared by more than ten tissues, including immune cells (*e.g.,* T cells, B cells), stromal cells (*e.g.,* fibroblasts, smooth muscle cells) and endothelial cells (*e.g.,* vascular and lymphatic endothelial cells).

To globally characterize the cell-type-specific chromatin accessibility landscape, we combined reads from cells of the same type and same tissue, performed peak calling, and iteratively merged the resulting peaks to a universal set of 1,341,077 open chromatin regions (±250 base pairs from peak summit; **Methods**). The genomic distribution of these peaks was largely consistent with previous sn-ATAC-seq profiling (14) (Fig. S3A): they predominantly located within introns (n=648,244; 48.3%) and intergenic regions (n=404,499; 30.1%), and to a lesser extent at TSS-promoter regions (n=34,487; 2.57%). These regions occupy 12.3% of the genome and intersect with 89.1% of the 339,815 CREs identified by the ENCODE consortium (20) (Fig. S3B). Using this universal peak set, cells of the same lineage clustered together across tissues, such as immune cells (*e.g.,* T/NK cells, B cells, plasma cells, macrophages) and stromal cells (*e.g.,* adipocytes, pericytes), further validating the accuracy of cell annotations (Fig. S3C).

Using an entropy-based method(12), we identified 320,304 cis-regulatory elements (CREs) that are cell-type-specific (Fig. S3D, Table S3; **Methods**). We next applied chromVar (21) to pinpoint TF motifs specific to each cell type (Fig. S3E). The results were consistent with established findings, highlighting the enrichment of *Runx3* in cytotoxic T cells and NK cells (22), *Sfpi1* in general myeloid cells (23), *Irf4* in plasma cells (24), and *Ebf1* in B cells and pericytes (25, 26) (Fig. S3E). The enrichment of these motifs was further validated by gene activity and cross-validated across different organs (Fig. S3F). Interestingly, we also observed TFs with a negative correlation between motif accessibility and gene activity, such as *Gfi1b* in erythroid cells (Fig. S3F), consistent with its role as a transcription repressor during erythroid development in humans(11, 27).

Next, we sought to use cell-type-specific accessible peaks to interpret genetic variants associated with complex traits and diseases. Specifically, we first mapped human SNPs to orthologous coordinates in the mouse genome and then applied linkage disequilibrium score regression (LDSC) to assess GWAS SNP enrichment within cell-type-specific accessible peaks, similar to previous studies(28) (**Methods**). Given that many GWAS SNPs reside in non-coding regions, this analysis provides insights into their functional relevance by prioritizing cell types of interest (Fig. S3G). For instance, Alzheimer’s disease SNPs were significantly enriched in microglia-specific CREs, consistent with prior studies(29). Meanwhile, SNPs associated with multiple sclerosis were enriched in B cells, T cells, and plasma cells(30, 31), while LDL-associated SNPs were linked to hepatocytes(32) and height-associated SNPs were linked to tenocytes. SNPs for type II diabetes showed the strongest association with pancreatic beta cells(33), and those for hypertension with juxtaglomerular cells in the kidney(34) (Fig. S3G).

### Age- and sex- dependent cell population change at the main cluster level

**Figure 2.**
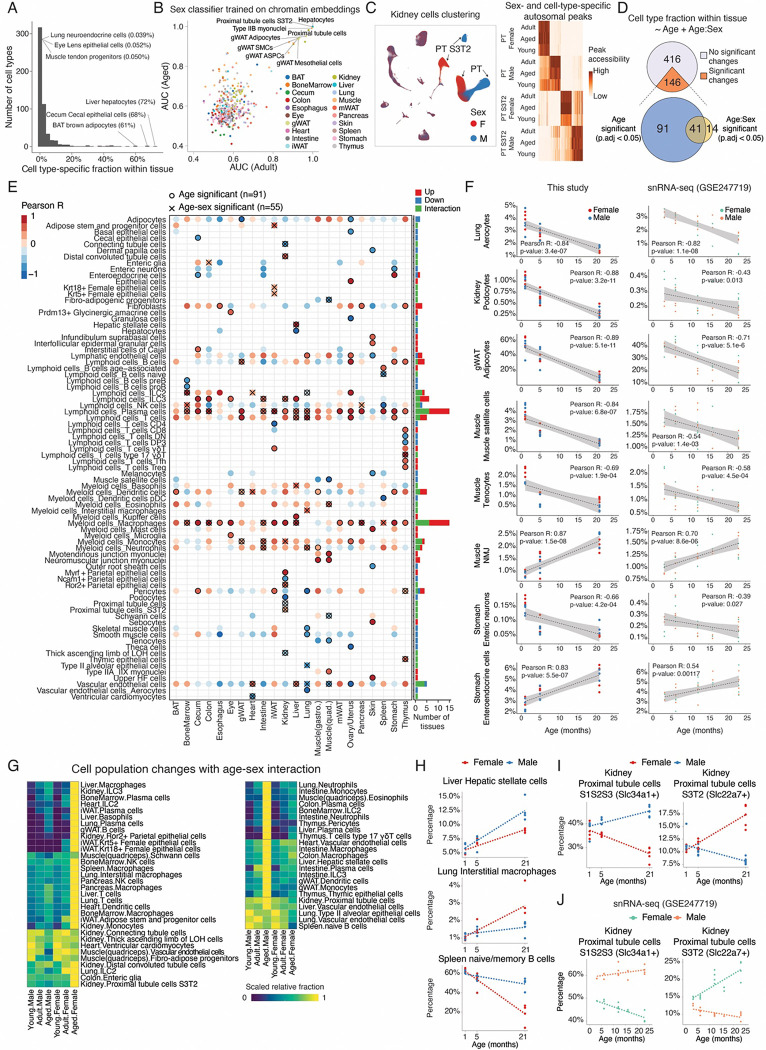
Age- and sex-dependent cell population changes at the main cell type level. **(A)** Histogram showing the distribution of cell type specific fractions within tissue. Only cells from adult mice (5-month-old) were used in this quantification. **(B)** K-means classifiers were trained to distinguish male and female cells for each main cell type using spectral embedding calculated by SnapATAC2 (17). Scatterplot comparing the area under the curve (AUC) values of models between adult and aged cells. Cell types with high sex dimorphism in both age groups are labeled. **(C)** Left: UMAP plots of all kidney cells, colored by sex (right), highlighting distinct chromatin states between sexes of proximal tubule cells (PT). Right: Heatmap displaying accessibility of autosomal peaks with sex- and cell-type-specific patterns, conserved across ages. **(D)** Venn diagram showing the number of main cell types whose relative proportions in corresponding tissues are significantly associated with age or age-sex interactions. **(E)** Left: Dotplot showing all significantly altered main cell types from (D) across tissues, colored by Pearson correlation between their relative proportion and age. Cell types with significant age or age-sex interactions are marked. Right: Barplot summarizing the number of tissues with significant changes for each cell type, colored by direction (up/down) or interaction. **(F)** Scatterplot illustrating examples of main cell types with significant age-related proportional changes (left), alongside validation using published snRNA-seq data (7) for the same cell type and tissue (right). **(G)** Heatmap showing relative proportions of main cell types with significant age-sex interactions, including female-biased (left) and male-biased (right) cell types. **(H)** Scatterplot highlighting cell types that change in the same direction with aging but exhibit sex-dependent magnitude. **(I-J)** Scatterplots of two kidney proximal tubule cell types with opposite sex-specific proportional trends **(I)** and snRNA-seq validation results **(J).**

By quantifying cell type abundances in tissues from 5-month-old mice, we observed significant variability across organs, with both highly abundant populations (*e.g.,* hepatocytes, cecal epithelial cells, brown adipocytes) and rare, organ-specific cell types detected (Fig. 2A). Notably, 43 organ-specific cell types constituted less than 1% of their tissue’s cellular composition, such as *Ascl1*+ neuroendocrine cells in the lung (0.039%) and *Gja8*+ lens epithelial cells in the eye (0.051%), underscoring the high sensitivity of scATAC-seq to identify low abundant but critical cell populations.

We next examined sex dimorphism for each main cell type. While most cell types exhibited similar population sizes between sexes (Pearson correlation r = 0.99, 0.98, 0.95 for young, adult and aged mice, respectively) (Fig. S4A), we identified a subset of cell types displaying strong sex-specific differences in chromatin states. Specifically, we trained k-nearest neighbor (KNN) classifiers to distinguish female and male cells of the same age for each cell type based on chromatin embeddings (**Methods**). We then used the area under the curve (AUC) metric to quantify classification accuracy, and cell types with AUC > 0.9 were considered to exhibit high sex-associated disparity (Fig. 2B). This approach revealed high sex-associated chromatin states in hepatocytes, proximal tubule cells, type IIB myonuclei, and multiple cell types from the gonadal white adipose tissue (*e.g.,* adipocytes, adipose stem and progenitor cells, mesothelial cells, and smooth muscle cells)(Fig. 2B), mirroring previous reports based on gene expressions (7, 35, 36). For example, proximal tubule (PT) cells in the kidney, including the general states spanning spatially connected segments (PTS1, PTS2 and PTS3; marked by *Slc34a1*) and a distinct state of segment 3 (PTS3T2, marked by *Slc22a7*) (37) was separated into clusters determined by sexes with unique marker gene accessibility (Fig. 2C and Fig. S4B). Furthermore, extensive changes underlying the sex difference were found on autosomal chromosomes and exhibited high cell-type-specificity (Fig. 2C, right).

We further investigated how aging influences the cell population dynamics across various tissues. Using linear regression, which included an age-sex interaction term, we pinpointed specific cell types that undergo significant changes with age within each organ (**Methods**). This analysis revealed that 146 organ-specific cell types are significantly altered upon aging (FDR of 0.05, R^2^ > 0.4), with 55 of these showing significant differences between males and females in aging (Fig. 2D–E; Table S4). Although aging-associated changes were noted in all profiled tissues, certain organs (*e.g.,* ovary/uterus, liver, thymus) exhibited a higher proportion of cell types with significant age-related changes (Fig. S5A), suggesting higher susceptibility to the aging effects in these organs.

A total of 62 main cell types were significantly expanded in aging regardless of sex (Fig. 2E, Fig. S5B top). Notably, the majority (68%) of these aging-expanded cell types are immune cells distributed across various organs (Fig. S5C). Plasma cells and macrophages were among the most significantly expanded, showing an increased relative proportion in 14 and 12 tissues, respectively, indicating a widespread increase across the organism (Fig. 2E). Several less characterized cell population changes were detected across multiple organs, such as the expansion of dendritic cells in the BAT and stomach, and ILC3s in the liver, colon, and cecum (Fig. S5D). Additionally, we observed the expansion of other immune cell types with more tissue-specific distributions, such as Kupffer cells in the liver and gamma-delta (γδ) T cells in the iWAT (Fig. S5D), consistent with previous studies(38–42). In addition to immune cells, we detected a notable expansion of various non-immune cell types during aging. For example, specialized myonuclei associated with neuromuscular junctions (NMJs) and myotendinous junctions showed increased abundance (Fig. 2E and 2F left), potentially acting as a compensatory mechanism to maintain muscle integrity and contractile function under age-related stress(43). Furthermore, we observed a rise in enteroendocrine cells within the stomach (Fig. 2E and 2F left), which could contribute to dysregulated digestive processes in aged individuals (44). Notably, these expansions were consistent across different sexes and were further validated by re-analysis of an independent single-cell transcriptome atlas of aging(7) (Fig. 2F, right).

The 29 aging-depleted cell types are predominantly tissue-specific (Fig. 2E, Fig. S5B bottom). This includes a notable reduction in immune cell progenitors such as pro-/pre-B cells in the bone marrow, which contributes to the age-related decline in immune function (45). Similarly, in the thymus, there is a decrease in immature T cells during the double-negative (DN) and double-positive (DP3) stages, indicative of diminished T cell production in the aging thymus (46). Other aging-depleted immune cell types, like NK cells and pDC cells in the spleen, were also detected, aligning with previous findings (47, 48) (Fig. 2E, Fig. S5B, S5E). Meanwhile, multiple non-immune cell types are significantly depleted across organs, especially in the ovary/uterus, skin, and lung (Fig. S6A). In the ovary/uterus, for example, granulosa cells and theca cells that collaborate in sex hormones production decline with age, together with basal epithelial cells and vascular endothelial cells. (Fig. 2E, Fig. S6B). This aligns with the decrease in ovarian follicles that characterizes reproductive aging (49, 50). In the skin, aging is associated with fewer melanocytes—a trend consistent with a 10–20% reduction per decade in individuals over 25–30 years of age (51)—as well as lower proportions of dermal papilla cells, outer root sheath cells, and lymphatic endothelial cells (Fig. 2E, Fig. S6B), the latter consistent with reduced skin lymphatic density observed in aging(52). Similarly, the aged lung exhibits declines in stromal cell types associated with blood vessels, including pericytes, smooth muscle cells (Fig. 2E, Fig. S6B), and a specialized endothelial cell type known as aerocytes(53) (Fig. 2F). These cells are important for maintaining proper vascular structure and function, suggesting that their depletion may contribute to compromised blood flow regulation in the elderly lung(54).

Moreover, we observed a decline in other functional cell types, such as kidney podocytes that filter primary urine from plasma (Fig. 2E–F), aligning with previous studies(55). The depleted cell populations also include a group of less characterized *Ncam1*+ parietal epithelial cells (Fig. 2E, Fig. S6B), which serve as progenitor cells capable of proliferation and differentiation into podocytes (56). The depletion of both mature podocytes and their precursor cells could contribute to the impaired capacity of the kidney to regenerate and maintain proper filtration over time. Additionally, aging-depleted cell types include liver hepatocytes (57), muscle tenocytes(58), muscle satellite cells (59), stomach enteric neurons, and adipocytes from multiple organs (*e.g.,* lung, gWAT, and ovary/uterus) (Fig. 2E–F and S5B), validated by re-analysis of external single-cell transcriptome dataset(7) (Fig. 2F). Interestingly, some of these aging-decreased cell types (*e.g.,* muscle satellite cells and tenocytes, bone marrow pro-/pre-B cells) exhibited reductions of more than 50% at relatively early stages (between 1 and 5 months) (Fig. 2F, S5B bottom, S6B), highlighting an early onset of aging impacts on the population of these cell types.

We further analyzed 55 cell types displaying significant age-sex interactions. Most sex-specific cell types (35 out of 55) are immune cells, including 19 that preferentially expanded in aging females and 15 in aging males (Fig. 2G). For instance, aged male mice showed significant increases in neutrophils (in the lung and intestine) and eosinophils in the muscle (Fig. 2G), consistent with male-specific expansions of these cells observed in prior human studies(60, 61). Conversely, aged females demonstrated increased populations of basophils (in the liver) and NK cells (in the pancreas and bone marrow) (Fig. 2G). Additionally, some cell types exhibited similar trends in both sexes but differed in the extent of change in different organs. For example, ILC2 cells in the bone marrow and hepatic stellate cells in the liver expanded more significantly in males, whereas the increase of interstitial macrophages in the lung and depletion of naive/memory B cells in the spleen was more pronounced in the female (Fig. 2G–H). Interestingly, certain cell types showed opposite trends between the sexes. For instance, the aforementioned specialized proximal tubule (PT) cell type (PT S3T2, characterized by *Slc22a7*) increased in females but decreased in males, whereas the other PT cells (PT S1S2S3, marked by *Slc34a1*) exhibited the opposite pattern (Fig. 2I). The findings were further validated by re-analysis of independent single-cell transcriptome atlas profiling both adult and aged tissues (7) (Fig. 2J).

### Age- and sex- dependent cell population change at the subtype level

**Figure 3:**
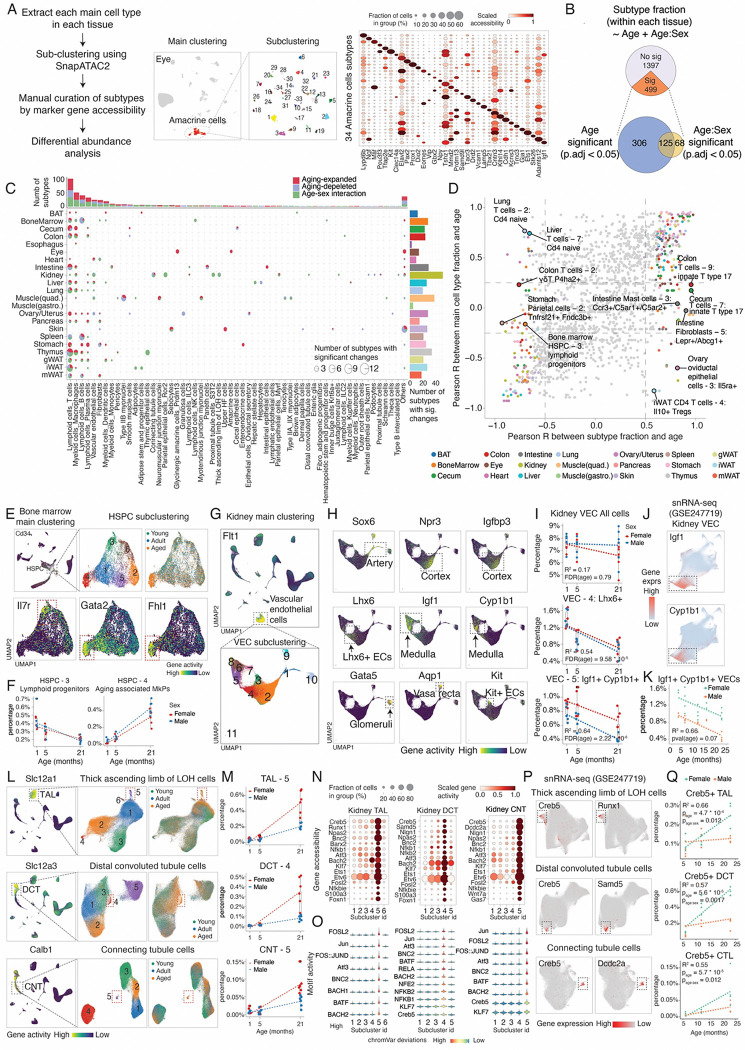
Age- and sex-dependent cell population change at the subtype level. **(A)** Left: schematic of the sub-clustering workflow. Middle: example UMAP plots showing sub-clustering analysis of eye amacrine cells, colored by subcluster ID. Right: dotplot displaying marker gene accessibility for amacrine cell subtypes. **(B)** Venn diagram showing the number of cell subtypes with significant changes associated with age or age-sex interactions. **(C)** Top: barplot showing the number of aging-associated subtypes for each main cell type. Center: dotplot summarizing the number aging-associated subtypes across main cell types and tissues. Right: barplot showing the number of aging-associated subtypes per tissue. **(D)** Scatterplot comparing aging-associated cellular fraction changes between aging-associated subtypes and their corresponding main cell types. Subtypes with distinct trends compared to their parent main cell types are labeled. **(E)** UMAP plots showing sub-clustering of bone marrow HSPCs to identify different subtypes, colored by subcluster ID (top middle), age group (top right), and marker gene accessibilities for lymphoid progenitor cells (bottom left) and age-associated megakaryocyte progenitors (bottom right). **(F)** Scatter plot showing cell proportion changes for lymphoid progenitor cells (HSPC-3) and age-associated megakaryocyte progenitors (HSPC-4), with a linear regression fit. Each dot represents one animal. **(G)** UMAP plot showing sub-clustering results of kidney vascular endothelial cells to identify different subtypes, colored by subcluster ID (bottom). **(H)** UMAP plots of kidney vascular endothelial cells, colored by the accessibility of genes that mark vascular cells across different spatial locations. **(I)** Scatter plots and boxplots showing the fractions of all kidney endothelial cells (top), vein endothelial cells (*Lhx6*+ VEC-4, center), and medulla endothelial cells (*Igf1*+ *Cyp1b1*+ VEC-5, bottom) across three age groups. **(J)** UMAP plots of vascular endothelial cells from a published snRNA-seq study (7), colored by the expression of genes marking medulla endothelial cells (as in **H**). **(K)** Scatterplot showing the aging-associated decline in the kidney medulla endothelial cells identified from a snRNA-seq atlas of aging(7). **(L)** UMAP plots showing all kidney cells (Left) and sub-clustering analysis (middle and right) for thick ascending limb cells (TAL, top), distal convoluted tubule cells (DCT, center), and connecting tubule cells (CNT, bottom), colored by main cell type specific markers (left), subtype ID (middle) and age group (right). Squares indicate the aging-associated cell subtypes. **(M)** Scatter plots showing the relative fractions of three reactive subtypes identified in (**K**) across ages, with separate linear regression fits for each sex. **(N)** Dotplots showing the gene marker accessibility marking the reactive states (TAL-5, DCT-4 and CNT-5) in each renal epithelial cell type. **(O)** Violinplots showing the motif accessibilities of transcription factors marking the reactive states in each renal epithelial cell type. **(P)** UMAP plots showing sub-clustering results for TAL, DCT, and CNT cells from published snRNA-seq data(7), colored by the expression of genes that mark reactive states (as in **N**). **(Q)** Scatterplots showing the relative fractions of reactive subtypes identified in **(P)** across aging, with separate linear regression fits for each sex.

We further explored cell population dynamics at the subtype level. First, we re-clustered each main cell type using *SnapATAC2(17)*, and manually curated cellular subtypes based on cluster-specific gene accessibility. Our analysis revealed significant heterogeneity within main cell types. For instance, amacrine cells in the eye, known for their extensive diversity(62), were clustered into thirty-four distinct subtypes (Fig. 3A). These subtypes were characterized by unique gene activity, peak accessibility, and TF motif enrichments (Fig. 3A, Fig. S7A–B), aligning with previous findings based on single-cell transcriptomic analyses (62). In total, our clustering analysis identified 1,828 subtypes from 386 main cell types across the entire organism, with a median of 1,610 cells per subtype and four subtypes per main cell type (Fig. S7C). Most subtypes (93.4%) were consistently detected across more than ten samples (Fig. S7C). Notably, we identified many rare cell subtypes that have not been extensively explored in prior chromatin accessibility studies. Notable examples include diverse enteroendocrine cells in the small intestine, including L cells (marked by *Gcg*, 0.092% of the total population), K cells (marked by *Gip*, 0.070%), D cells (marked by *Sst*, 0.028%), enterochromaffin cells (marked by *Tph1*, 0.084%), and enteroendocrine progenitor cells (marked by *Neurog3*, 0.081%) (Fig. S7D–S7E).

We next investigated cell population changes at the subtype level. Using the same analytical pipeline for identifying aging-associated main cell types, we identified 499 subtypes (out of 228 organ-specific main cell types) with significant cell population dynamics associated with aging (FDR of 0.05, R^2^ > 0.4) (Fig. 3B, Table S5). Of these, 193 subtypes exhibited significant sex-specific aging differences (FDR of 0.05) (Fig. 3C). A median of 24 significantly altered subtypes was identified per tissue, ranging from 51 in the kidney to 3 in the esophagus (Fig. 3C). While the dynamics of aging-associated subtypes were generally consistent with their main cell type changes (Pearson r: 0.72, p-value < 2.2 * 10^−26^), distinct trends emerged in certain subtypes compared to their parent main cell types (Fig. 3D). For instance, specific aging-associated subtypes such as *Lepr1+ Abcg1+* fibroblasts and *Ccr3+ C5ar1+ C5ar2+* mast cells expanded in the aged intestine, while their main cell types remained relatively stable with age (Fig. S7F–G). In the liver and lung, while the overall main T cell population increased in aged tissues, subtypes corresponding to naive T cells were depleted (Fig. S7F–G). In contrast, we observed an expansion of a subtype corresponding to *Il10*+ *Foxp3*+ regulatory T cell in the iWAT, despite the overall CD4 T cell proportion decreasing with aging (Fig. S7F–G). We also observed opposing aging-associated dynamics of subtypes from the same cell type. For example, in the bone marrow hematopoietic stem and progenitor cells (HSPCs), there is a marked decrease in lymphoid progenitors (HSPC-3, marked by *Il7r (63)*), accompanied by a significant expansion of aging-associated megakaryocyte progenitors (HSPC-4, marked by *Gata2, Fhl1, and Hoxb5*) (Fig. 3E–F), consistent with findings from a recent study(64).

Several aging-associated subtypes correspond to the same cell type in distinct spatial locations, uncovering region-specific vulnerabilities during aging. For example, renal vascular endothelial cells (ECs) can be clustered into heterogeneous subtypes representing ECs from different anatomical locations within the nephron(65), including the artery (*Sox6+*), cortex (*Npr3+, Igfbp3+*), vein (*Lhx6+*), medulla (*Igf1+, Cyp1b1+*), vasa recta (*Aqp1+*), and glomeruli (*Gata5+*) (Fig. 3G–H). Interestingly, while the total abundance of ECs remains relatively unchanged at the main cell type level, we observed a pronounced depletion of two EC subtypes—*Lhx6+* vein ECs and *Igf1+, Cyp1b1+* medulla ECs (Fig. 3I)—indicating that endothelial cells in these specific regions are more vulnerable to aging. The findings were further validated through a published snRNA-seq dataset(7): we observed a consistent decline of *Igf1*+ *Cyp1b1*+ medulla ECs across aging in both sexes, despite a higher baseline proportion in females (Fig. 3J–K).

Meanwhile, certain aging-associated cell subtypes across different spatial locations share similar molecular features. For example, sub-clustering analysis of three specialized renal epithelial cell types—the thick ascending limb of the Loop of Henle (TAL), distal convoluted tubule (DCT), and connecting tubule (CNT)—revealed three subtypes (*i.e.,* TAL-5, DCT-4, and CNT-5) that exhibit significant expansion in aging (Fig. 3L–M), particularly in females. All three subtypes converged into a reactive aging-associated epithelial cell state that is characterized by the activation of transcription factors linked to inflammation and stress responses (*e.g., Creb5, Bnc2, Runx1, Bach2, Fosl2,* and *Klf7*), confirmed through gene body accessibility (Fig. 3N) and TF motif activity (Fig. 3O). We further validated the existence of these states (Fig. 3P) and female-biased expansions (Fig. 3Q) by re-clustering the same cell type from a single-cell transcriptomic atlas of aging(7). The emergence of this reactive cellular state underscores the potential influence of inflammatory signaling on epithelial cells specifically in the aged female kidney. In summary, our subtype-level analysis revealed both region-specific and shared aging-associated changes.

### Aging-associated population dynamics of broadly distributed immune subtypes

We next examined whether broadly distributed cells displayed similar dynamics across multiple organs in aging. We first analyzed immune cells, including T cells (n = 1,264,322 cells), B cells (n = 1,079,641 cells), plasma cells (n = 157,545 cells), innate lymphoid cells (NK cells, ILC1, ILC2 and ILC3) (n = 87,550 cells), monocytes and macrophages (n = 581,877 cells), and dendritic cells (n = 306,562 cells) isolated from various organs.

**Figure 4.**
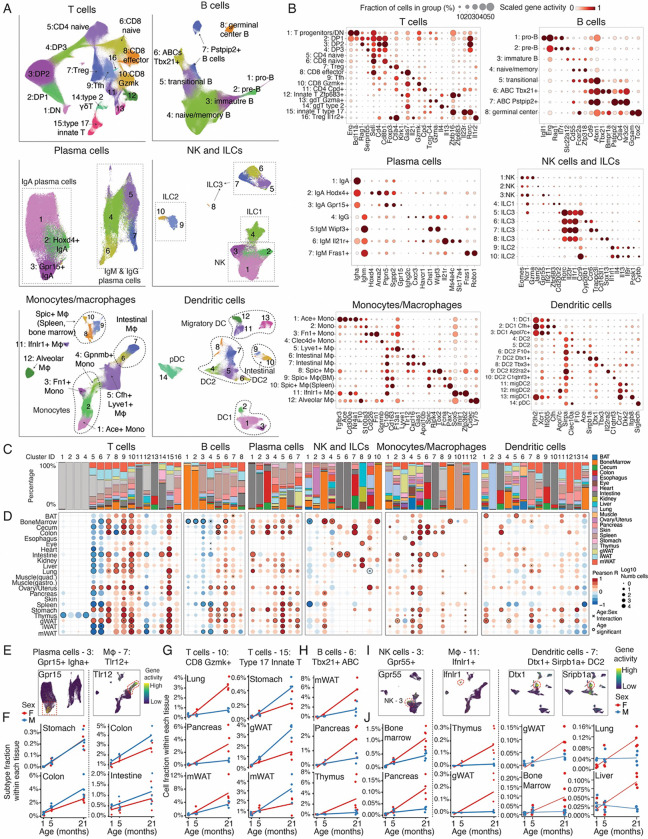
Aging-associated dynamics of broadly distributed immune subtypes. **(A)** UMAP plots showing combined clustering results for T cells, B cells, plasma cells, innate lymphoid cells, macrophages and dendritic cells across tissues, colored by subtype id. **(B)** Dotplots showing accessibilities of genes used for annotating immune cell subtypes. **(C)** Stacked bar plots showing the tissue composition for each immune subtype. **(D)** Dotplots showing the proportional changes of each immune subtype in each tissue across aging, colored by Pearson correlation between their relative proportion (within each tissue) and age. Subtypes with significant age or age-sex interactions are marked. **(E)** UMAP plots highlighting aging-expanded subtypes of plasma cell (left) and macrophage (right) in gastrointestinal tissues, colored by marker gene accessibility (*Gpr15* for plasma cell-3 and *Tlr12* for macrophage-7). **(F)** Scatterplot showing the relative fractions of *Gpr15+ Igha+* plasma cells and *Tlr12*+ macrophages along aging. **(G)** Scatterplots showing the relative fractions of *Gzmk+* CD8 T cells (left) and *Il23r+ Zbtb16*+ type 17 innate T cells (right) along aging, with separate linear regression fits for each sex. **(H)** Scatterplot showing the relative fractions of *Tbx21+* aging-associated B cells along aging, with separate linear regression fits for each sex. **(I)** UMAP plots showing subtypes exhibiting female-biased expansion along aging, colored by accessibilities of marker genes. (*Gpr55 for NK-3*, *Ifnlr1* for macrophage-11, *Dtx1* and *Sirbp1a* for dendritic cells-7). **(J)** Scatterplot showing the relative fractions of *Gpr55+* NK cells (left), *Ifnlr1*+ macrophages (middle) and *Dtx1+ Sirbp1a+* type 2 dendritic cells (right) along aging, with separate linear regression fits for each sex.

Clustering analysis for each cell type revealed a total of sixty-seven highly heterogeneous immune cell subtypes, each defined by distinct chromatin accessibility patterns at key marker genes (Fig. 4A–B). For instance, the combined clustering of T cells yielded sixteen subtypes spanning a differentiation trajectory from T progenitor/double-negative (DN) cells, through double-positive (DP) cells, and onward to mature T cell states (Fig. 4A–B, top left), including CD4 T cells (*Cd4*+), CD8 T cells (*Cd8b1*+), γδ T cells (*Tcrg-C4*+), and innate-like T cells (*Zbtb16*+) (Fig. S8A). Similarly, we identified eight B cell clusters forming a trajectory from pro-B and pre-B cells (*Erg+, Rag1+, Il7r+*) through naive/memory B cells (*Cd55+, Fcer2a+*) and finally transitioned into aging-associated states marked by either *Tbx21* or *Pstpip2* (Fig. 4A–B, top right; Fig. S8B). These aging-associated B cell states closely mirror recent single-cell transcriptomic findings(66). Within the plasma cell population, we detected IgA, IgM, and IgG plasma cells, each comprising several distinct subtypes (Fig. 4A–B, middle left). For innate lymphoid cells, ten clusters were identified, including multiple subtypes within NK cells, ILC2, and ILC3 (Fig. 4A–B, middle right; Fig. S8C). Monocytes and macrophages were resolved into twelve subtypes (Fig. 4A–B, bottom left), capturing the transition from *Ace*+ monocytes to *Lyve1*+ tissue-resident macrophages, and various specialized macrophages across different tissues. Dendritic cells (DC) were categorized into fourteen subtypes (Fig. 4A–B, bottom right), including *Xcr1*+ type I DCs, *Sirpa*+ type II DCs, *Ccr7*+ migratory DCs, and *Siglech*+ plasmacytoid DCs, with diverse subsets observed within each major type.

We then quantified the tissue composition of each immune subtype. Although many subtypes were detected globally, certain subtypes exhibited high organ specificity (Fig. 4C). For example, immature T cells (T cells −1, T cells - 2, T cells −3, and T cells - 4) were predominantly in the thymus, while *Gzma+* γδ T cells (T cells - 13) were mainly localized to the intestine, cecum, and colon(Fig. 4C). Similarly, B cell progenitors (B cells - 1 and B cells - 2) were primarily found in the bone marrow, and IgA+ plasma cells (plasma cells - 1, plasma cells −2, and plasma cells - 3) were enriched in gastrointestinal organs (*e.g.,* intestine, cecum, and colon)(Fig. 4C). For macrophages (MAC), *Cxcl16*+ *Tlr12*+ macrophages (MAC-6 and MAC-7) were constrained to the small and large intestine, while *Spic*+ macrophages were unique to bone marrow (MAC-9) and spleen (MAC-10)(Fig. 4C). The latter aligns with previous reports about the role of *Spic* in the development of iron-recycling macrophages in these two organs(67). Certain dendritic cells also demonstrated tissue-specific characteristics, such as *Apol7c*+ type I DCs (DC-3), *Il22ra2*+, and *C1qtnf3*+ type II DCs (DC-9 and DC-10), which were exclusively identified in the gut(Fig. 4C).

We next assessed how these immune subtypes changed with aging (Fig. 4D). Six immune subtypes were found significantly depleted across multiple tissues, including naive CD4+ T cells in ten tissues, naive CD8+ T cells in five tissues, and immature B cells in the bone marrow, spleen, and mWAT(Fig. 4D). We also observed a significant depletion of organ-specific immune progenitor cells, such as immature T cells in the thymus and pro-B/pre-B cells in the bone marrow, which subsequently led to a reduction of naive/memory B cells in tissues like the spleen and mWAT(Fig. 4D). In addition, we uncovered less-characterized immune cell dynamics validated across multiple organs. For instance, *Zfp683+* tissue-resident memory T cells were consistently depleted in the intestine, iWAT, and mWAT (T cells - 12, Fig. 4D), and *Gzma+* resting NK cells (68) were depleted in both the spleen and bone marrow (NK - 1; Fig. 4D). Notably, the depletion of this resting NK cell subtype occurred concurrently with an expansion of a *Gpr55+ Ifi211+* proinflammatory NK cell subtype (69) (NK - 3) in the same organs (Fig. 4D).

In contrast to these depletions, the majority of immune subtypes (42 out of 67) significantly expanded during aging in at least one tissue (Fig. 4D). These expansions included nine T cell subtypes, eight NK and ILC subtypes, five B cell subtypes, seven dendritic cell subtypes, and nearly all plasma cell and monocytes/macrophages (‘MoMa’) subtypes (Fig. 4D). Notable examples of broadly expanding populations included *Zbtb16+ Il23r+* type 17 innate T cells (T cells - 15), *Gzmk+* CD8 T cells (T cells - 10), *Il21+* follicular helper T cells (T cells - 9), *Foxp3+* regulatory T cells (T cells - 7), *Tbx21+* aging-associated B cells (B cells - 6), *Chst1+ Wipf3+* IgM plasma cells (plasma cells - 5), *Clec4d+* monocytes (MoMa - 4), and *F13a1+ Lyve1+* macrophages (MoMa - 5), each of which expanded in multiple organs (Fig. 4D). Beyond these widespread patterns, some aging-associated expansions were confined to specific organs. For example, several immune subtypes expanded preferentially in the gastrointestinal tract, such as *Gpr15+ Stc2+* IgA plasma cells (plasma cells - 3; Fig. 4E–F, left), *Tlr12+* macrophages (macrophages - 7; Fig. 4E–F, right), *Ccr6+ Trappc3l*+ ILC3 cells (NK/ILC - 7), and *Apol7c*+ type I DCs (dendritic cells - 3) (Fig. 4D). We also identified tissue-specific expansions of *Cd163I+ Sox13+* ILC3 cells (NK/ILC - 8) in the eye, kidney, and liver, as well as *Pcsk1+* ILC2s (NK/ILC - 9) in the ovary/uterus and *Cybb+* ILC2s (NK/ILC - 10) in the bone marrow (Fig. 4D). Interestingly, some immune subtypes exhibited opposite trends depending on the organ. For instance, *Klrk1+ Gas7+* CD8 effector T cells (T cells - 8) expanded in the liver and lung but were depleted in gastrointestinal tissues (*e.g.,* colon and cecum) (Fig. 4D), potentially due to distinct local microenvironmental cues that shape tissue-specific aging dynamics.

Aging-associated immune cell dynamics also differed notably between males and females. For example, *Gzmk+* CD8 exhausted T cells exhibited greater expansion in females (Fig. 4G, left), notably in the lung, pancreas, and mWAT, while type 17 innate T cells showed a stronger increase in males, especially in the stomach, gWAT, and mWAT (Fig. 4G, right). Overall, however, we observed that females tend to have a greater expansion of immune cell subtypes than males during aging (53 female-biased subtypes compared to 31 male-biased subtypes, Fig. S8D), including *Tbx21+* aging-associated B cells (in mWAT, pancreas, thymus; Fig. 4H), *Gpr55+ Ifi211+* NK cells (in bone marrow, pancreas, iWAT, spleen; Fig. 4I–J, left), *Sox5+ Ifnlr1+ Zfp362+* macrophages (in thymus, gWAT; Fig. 4I–J, middle) and *Dtx1*+ *Sripb1a*+ type II dendritic cells (Fig. 4I–J, right). These subtypes potentially contribute to the higher prevalence of autoimmune diseases in women(70). Interestingly, we observed an enrichment of male-biased subtypes within gastrointestinal tissues, with 15 out of 31 originating from the esophagus, stomach, intestine, cecum, and colon, compared to only 2 out of 53 in female-biased ones (Fig. S8D). Noteworthy examples include the expansion of *Ccr9*+ ILC3s (NK/ILC-5) and *Cyp26b1*+ ILC3s (NK/ILC-6) in the intestine (Fig. S8D), reflecting more pronounced changes in the innate immunity during male intestinal aging (71).

### Aging-associated population dynamics of broadly distributed non-immune subtypes

**Figure 5.**
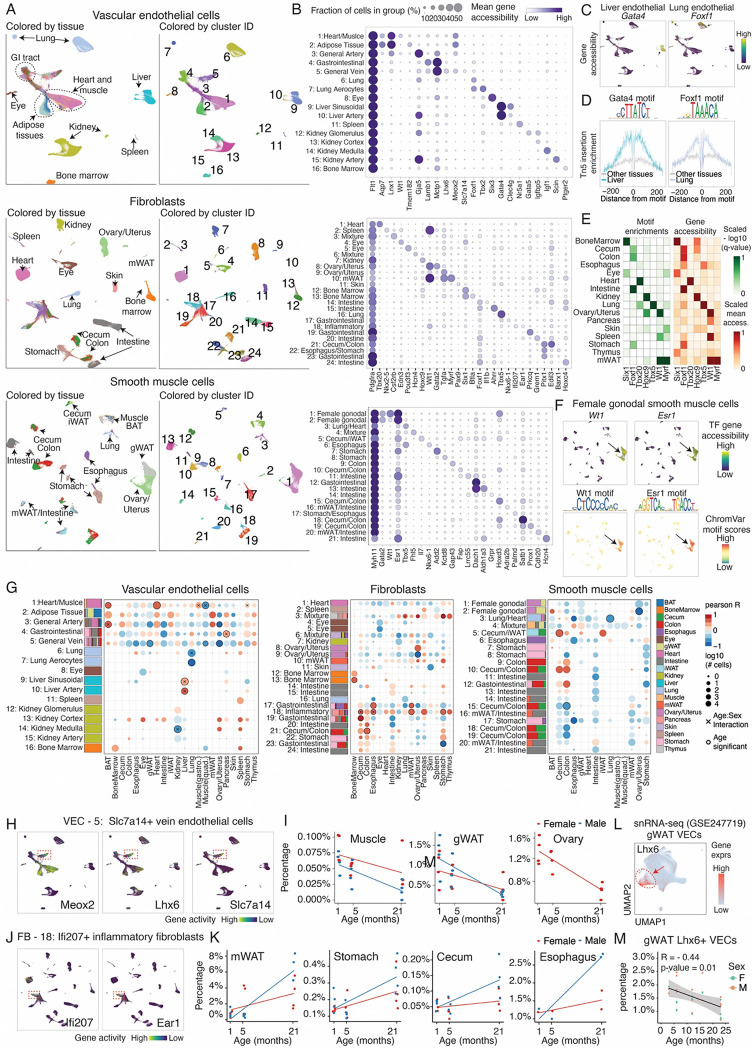
Aging-associated dynamics of vascular endothelial cells, fibroblasts, and smooth muscle cells across organs. **(A)** UMAP visualization showing the combined clustering for vascular endothelial cells (VECs, top), fibroblasts (FBs, middle) and smooth muscle cells (SMCs, bottom) across tissues, colored by tissue origin (Left) and cluster ID (Right). **(B)** Dot plots showing the accessibility of marker genes for cell clusters of VEC (top), FB (middle) and SMC (bottom), including a general marker for each cell type (*Flt1* for VEC, *Pdgfra* for FBs, *Myh11* for SMCs) **(C)** UMAP plot of VECs colored by accessibilities of the liver VEC marker *Gata4* and lung VEC marker *Foxf1* **(D)** Motif footprinting analysis showing enrichment of GATA4 motifs in liver VECs and FOXF1 motifs in lung VECs compared with other tissues. **(E)** Heatmaps showing the motif enrichments and gene body accessibility of tissue-specific transcription factors. Motif enrichment scores (−log10 q-value) were computed with HOMER (73) using tissue-specific peaks and scaled to the top enriched tissue. **(F)** UMAP plots of SMCs colored by gene accessibility (Top) and chromVar motif deviation scores (Bottom) for gonadal SMC markers *Wt1* and *Esr1*. **(G)** The stacking bar plots show tissue composition per cluster for VECs (Left), FBs (Middle), and SMCs (Right). The dot plots show aging-associated proportional changes of clusters across tissues, colored by Pearson correlation between cluster proportion (normalized per tissue) and age. Clusters with significant age/age-sex effects are annotated (p-adjusted < 0.05). **(H)** UMAP of VECs colored by the accessibility of VEC-5 markers (*Meox2*, *Lhx6*, *Slc7a14*). **(I)** Scatterplots showing the age-related decline in VEC-5 proportions in muscle (quadriceps), gonadal white adipose tissue (gWAT), and ovary/uterus. **(J)** UMAP of FBs colored by the accessibility of FB-18 markers (*Ifi207*, *Ear1*). **(K)** Scatterplots showing the age-associated increase in FB-5 proportions in mesenteric WAT (mWAT), stomach, cecum, and esophagus. **(L)** UMAP plot showing gWAT VEC cells isolated from snRNA-seq data (7), colored by *Lhx6* expression (VEC-5 marker). **(M)** Scatterplot showing age-dependent reduction in *Lhx6*+ gWAT endothelial cells.

Next, we expanded our analysis to aging-associated dynamics in non-immune cell populations, focusing on vascular endothelial cells (VECs) (n = 426,160 cells, Fig. 5A–B, top), fibroblasts (n = 377,181 cells, Fig. 5A–B, middle), and smooth muscle cells (n = 101,284 cells, Fig. 5A–B, bottom), which are broadly distributed across organs. Unlike immune cells that share molecular states across tissues, these non-immune cell populations displayed remarkable organ specificity, with most (34 out of 61) clusters dominated by a single tissue (>90% of cells from one tissue). For example, we identified VECs specific to the liver (marked by *Gata4*) and the lung (marked by *Foxf1*) (Fig. 5C). The tissue specificity was further confirmed through motif footprinting analyses of marker TFs (Fig. 5D), and consistent with previous study (72). Similarly, tissue-specific TFs were observed in different fibroblast populations: *Six1* in bone marrow fibroblasts, *Foxf1* in gastrointestinal fibroblasts, *Tbx20* in cardiac fibroblasts, *Hoxc9* in kidney fibroblasts, and *Tbx5* in lung fibroblasts (Fig. 5E). We also identified subtypes exhibiting sex specificity. For instance, we identified subtypes of smooth muscle cells unique to female gonadal organs (*e.g.,* ovary, uterus, and gWAT). These cells were marked by the transcription factors *Wt1* and *Esr1,* supported by both gene accessibility and motif activity (Fig. 5F).

We further examined how these broadly distributed non-immune subtypes changed with aging (Fig. 5G). Compared to immune cells that overall expand across organs, these broadly distributed non-immune cell types exhibited strong tissue-specific changes during aging (Fig. 5G). For example, we observed an age-associated depletion of various organ-specific endothelial subtypes, such as cells in the kidney (VEC-14, *Igf1*+ kidney medulla endothelial), lung (VEC-7, *Tbx2*+ lung aerocytes), and muscle (VEC-1, *Aqp7*+) (Fig. 5G and S9A). By contrast, organ-specific endothelial cells in the liver (VEC-9, *Clec4g*+ sinusoidal endothelial) and pancreas (VEC-4, *Lamb1*+ VECs) expanded significantly during aging (Fig. 5G and S9B). The expansion of liver sinusoidal endothelial cells aligns with previous findings from *Tabula Muris Senis(57)*. Fibroblasts (FB) likewise displayed tissue-specific aging trajectories, showing expansion in aged bone marrow (*Btla*+ FB-13), colon (*Edil3*+ FB-21), cecum (*Prkcq*+ FB-19), stomach (*Csf2rb*+ FB-3), and mWAT (*Nkx6–1*+ FB-17) (Fig. 5G and S9C). In contrast, two fibroblast subtypes (ovary *Tgfa*+ FB-9 and stomach *Sox6*+ FB-23) significantly decreased in aging (Fig. 5G and S9D). For smooth muscle cell (SMC) subtypes, there is a strong depletion of several subtypes across organs, such as colon (*Hoxd3*+ SMC-15), lung (*Tbx5*+ SMC-3), and ovary/uterus (*Gata2*+ SMC-2) (Fig. 5G and S9F). In the cecum, however, there was an increased population of SMCs marked by inflammatory gene markers such as *Il7* and *Serpina1c* (SMC-5, Fig. 5G and S9G).

Importantly, we identified several cell subtypes that shared similar aging dynamics across multiple tissues. For example, a *Slc7a14*+ *Lhx6*+ *Meox2*+ vein endothelial cell subtype (VEC-5) was significantly depleted in several aged tissues (*e.g.,* muscle, gWAT, and ovary/uterus) (Fig. 5H–II), which could contribute to the endothelium dysfunction in aging. Likewise, an aging-associated fibroblast subtype characterized by elevated inflammatory markers (*e.g., Ifi207*, *Ifi205*, *Ear1*) expanded in several aged organs (*e.g.,* mWAT, stomach, cecum, esophagus) in a male-specific manner (Fig. 5J–K). Of note, these aging-associated subtype dynamics were validated through external datasets (7), such as the depletion of *Lhx6*+ endothelial cells in gWAT (Fig. 5L–M) and the expansion of *Csf2rb*+ fibroblasts in the stomach (Fig. S9E).

### Age- and sex-dependent epigenetic signatures of aging

**Figure 6.**
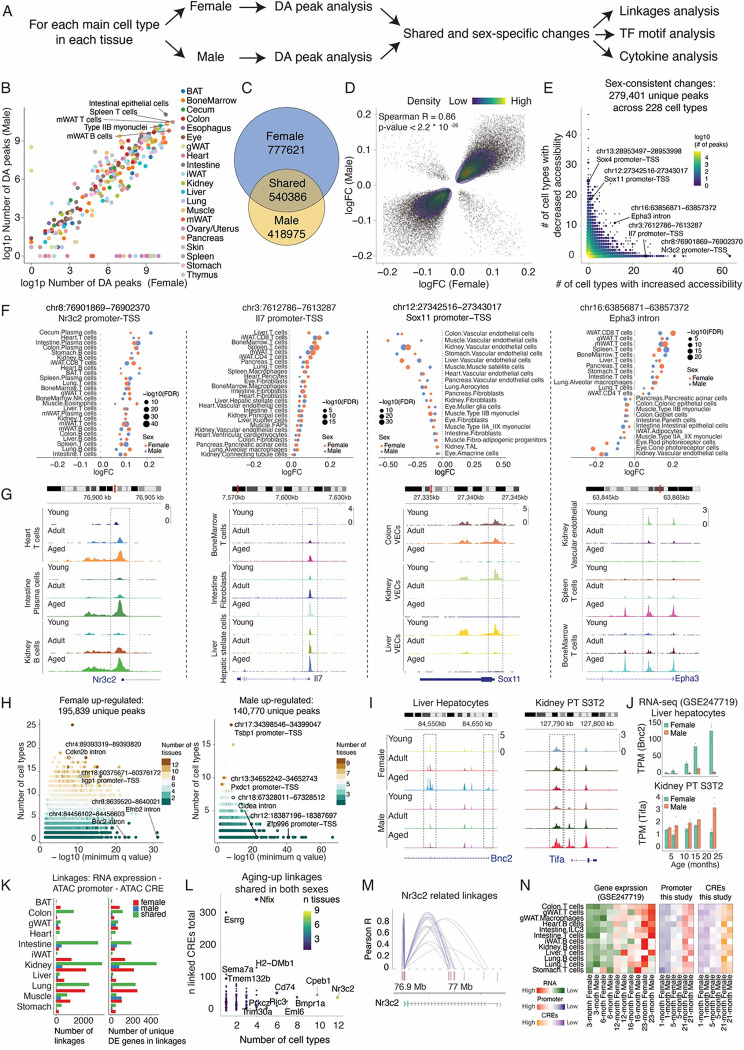
Aging-associated changes of chromatin accessibility. **(A)** Scheme for computational analyses to identify differentially accessible (DA) peaks along aging, their putative gene targets, and upstream regulators. **(B)** Scatterplot comparing the number of DA peaks between males and females for each main cell type. Cell types with a high number of DA peaks are labeled. **(C)** Venn plot of cell-type-specific DA peaks shared or unique to males and females. **(D)** Correlation of log transformed fold change (logFC, aging-associated accessibility changes per month, calculated via edgeR (77)) for DA peaks shared between sexes. **(E)** Scatterplot summarizing DA peaks with concordant changes with aging in both sexes. Axes show counts of cell types with increased (x-axis) or decreased (y-axis) accessibility; color denotes peak density. **(F)** Dot plots showing log transformed fold change (logFC) for DA peaks with consistent increases (promoters of *Nr3c2* and *Il7*), decreases (promoters of *Sox4*), or divergent trends (intronic peak of *Epha3*) across cell types. **(G)** Genomic tracks visualizing the accessibilities of DA peaks in (**F**) across three age groups. **(H)** Dot plots of sex-specific DA peaks for females (Left) and males (Right). Y-axis: number of cell types with significant increases; x-axis: −log10(q-value) for the most significant cell type. Color denotes the number of tissues with significant increases. **(I)** Genomic tracks of female-specific DA peak in liver hepatocytes (Left) and male-specific DA peak kidney proximal tubule cells S3T2 (Right). **(J)** Barplot showing validation of sex-specific chromatin accessibility changes using gene expression data (7) of the same genes in the same cell type as in (**I**). Dots represent gene expression of target genes across independent animals, quantified by transcripts per million (TPM). **(K)** Barplot showing the number of cell-type-specific, aging-associated gene-promoter-CRE linkages (Left) and the number of unique genes within those linkages (Right) for each tissue. **(L)** Dot plot of genes with aging-upregulated linkages in both sexes. X-axis: cell-type count; y-axis: CRE count per gene. Color denotes the number of tissues containing cell types with significant increases. **(M)** Genome browser plot showing links between putative cis-regulatory elements and promoters for *Nr3c2*. **(N)** Heatmaps showing coordinated increases in gene expression, promoter accessibility, and linked distal CRE accessibility of *Nr3c2* across multiple immune cells with aging.

We next investigated the organism-wide epigenetic aging program by examining 349 organ-specific main cell types. For each major cell type in each tissue, we performed differential accessibility (DA) analyses in females and males separately (**Methods**). From these analyses, we identified both shared and sex-specific age-associated alterations in chromatin accessibility. We then carried out linkage analysis to map downstream genetic targets of these chromatin sites and examined upstream regulators by assessing the activity of intrinsic transcription factors and extrinsic cytokine signaling pathways that drive epigenetic reprogramming during mammalian aging (Fig. 6A).

In total, we identified 1,318,007 and 959,361 cell-type-specific and aging-associated DA peaks in females and males, respectively (Fig. 6B, Fig. S10A, Table S6–S8). While the number of DA peaks generally correlated with cell numbers, a few cell types exhibited notably strong aging-related chromatin changes, standing out as outliers with high regression residuals—such as B cells from brown adipose tissue or kidney, tenocytes, and muscle satellite cells (Fig. S10B). Among the identified DA peaks, a total of 540,386 cell-type-specific DA peaks were present in both sexes, most (96.9%) exhibiting consistent changes in the same cell type across both males and females (Spearman correlation of log transformed fold change = 0.86, p-value < 2.2 * 10^−16^, Fig. 6C–D).

While many DA peaks were restricted to only a few cell types (91.4% found in no more than 3 cell types), we also uncovered a subset of peaks (n = 803) that were consistently altered across many cell types (n >= 10) and tissues (Fig. 6E). This subset highlights coordinated chromatin dynamics across diverse cell types and regions during mammalian aging. Notable examples include increased promoter accessibility of the mineralocorticoid receptor *Nr3c2* (in 63 cell types, Fig. 6F–G, left) and the proinflammatory cytokine *Il7* (in 26 cell types, Fig. 6F–G, middle left), predominantly within immune cells. Increased *Nr3c2* activity also marked the aging-associated B cell states (Fig. 4B). Conversely, we identified DA peaks with decreased accessibility in aging, such as promoters of *Sox4* (in 25 cell types) and *Sox11* (in 19 cell types)(Fig. 6E). Specifically, *Sox11*, a transcription factor involved in angiogenesis (74), showed reduced accessibility in endothelial cells across multiple organs (Fig. 6F–G, middle right), which could contribute to the global functional decline of the vascular system.

Aging-associated chromatin alterations were not always uniform across cell types. For example, 131 peaks showed opposite changes in different cell types (at least five cell types in each direction). One example is a peak in the intron of *Epha3*, which displayed increased accessibility in T cells and macrophages across 12 tissues but decreased accessibility in non-immune cell types such as vascular cells, neurons, and epithelial cells (Fig. 6F–G, right, Fig. S10C). These cell-type-specific patterns were confirmed across both sexes (Fig. 6F), suggesting that aging-associated chromatin alterations are shaped by unique cellular contexts and lineage-specific regulatory networks.

These epigenetic changes in aging also vary by sex. Considering peaks with significantly increased accessibility in aging, we found 195,893 sex-specific peaks in females and 140,770 in males (Fig. 6H). Top female-specific DA peaks validated across multiple cell types from different tissues included intronic peaks at the senescence-associated gene *Cdkn2b* (in 18 cell types) and promoter peaks of the interferon-inducible gene *Ligp1* (in 14 cell types) and *Ifi211* (in 16 cell types) (Fig. 6H). Similarly, we observed male-specific DA peaks shared across multiple cell types, such as the increased promoter accessibility of *Tsbp1* across 18 cell types (Fig. 6H). We also identified cell-type-specific chromatin accessibility changes that were highly sex-dependent. For example, aged hepatocytes in females showed up-regulation of peaks overlapping with *Bnc2* (Fig. 6I, left), a gene involved in extracellular matrix production and degradation (75). In contrast, aged hepatocytes in males showed up-regulation of peaks overlapping with *Cidea* (Fig. S6D, left), a gene involved in lipid particle organizations. Similarly, in a subtype of proximal tubule cells segment 3 (PT S3T2), peaks overlapping *Mill2* were more accessible in aged females (Fig. S6D, right), whereas intronic peaks in *Tifa*—a gene associated with programmed cell death (76)—were more accessible in aged males (Fig. 6I, right). Consistent with these findings, this cellular state specifically declined in aged males (Fig. 2H). Notably, these sex- and cell-type-specific peaks were further validated by corresponding changes in promoter accessibility and gene expression during aging (Fig. 6I–J and S6E).

To infer putative target genes of aging-associated chromatin alterations in cis-regulatory elements (CRE), we linked non-promoter DA peaks to nearby promoters based on their co-accessibility across samples for each cell type, thus generating promoter-CRE linkages. We then integrated the data with a published snRNA-seq atlas of aging (7) to identify genes exhibiting consistent changes between promoters and expressions with age (**Methods**). This analysis identified 21,360 and 14,564 cell-type-specific gene-promoter-CRE linkages in males and females, respectively, with 12,862 linkages shared by both sexes (Table S9). In total, this revealed 15,090 CREs linked to aging-associated changes in 2,001 differentially expressed (DE) genes across 86 organ-specific cell types (Fig. 6K). Collapsing sex-shared, up-regulation linkages across cell types revealed concordant changes supported in three molecular evidences (i.e, genes, promoters and putative enhancers) (Fig. 6L). One notable example is *Nr3c2*, linked to 25 CREs, most located in intronic regions (Fig. 6M). These CREs showed strongly increased accessibility during aging across multiple immune cell types and tissues, in line with changes in promoter accessibility and gene expression from the same cell type (Fig. 6N).

### Intrinsic and extrinsic regulators of epigenetic reprogramming in mammalian aging

**Figure 7.**
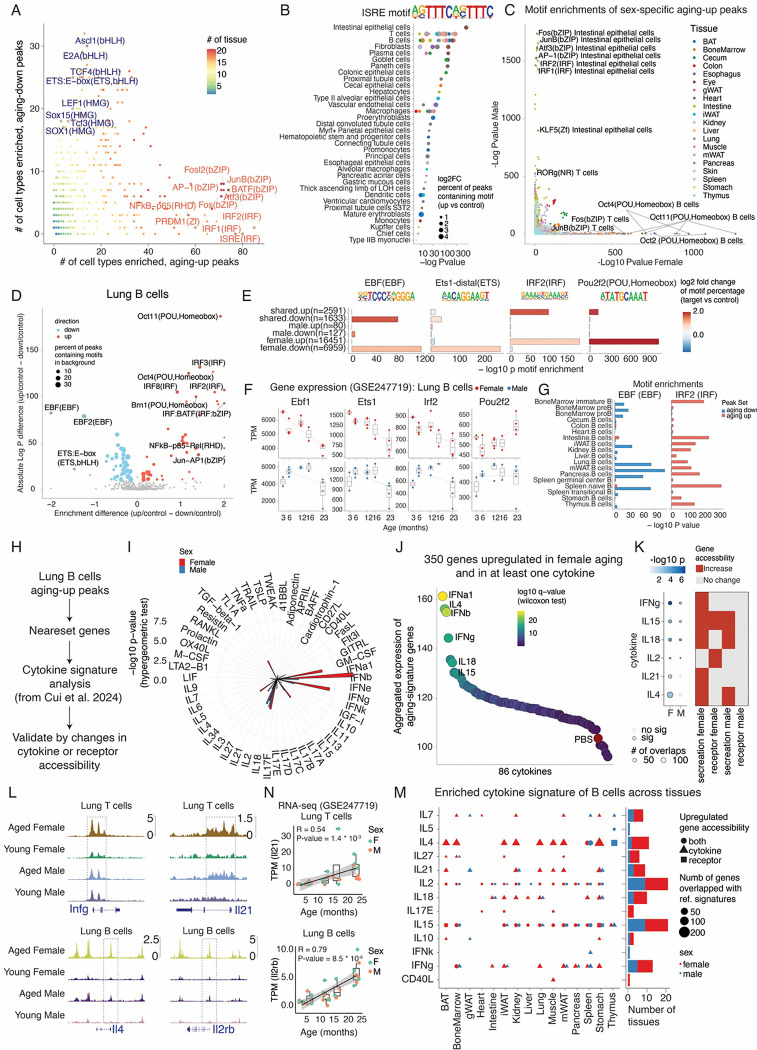
Identifying upstream regulators of aging-associated chromatin changes. **(A)** Sex-shared aging-associated up-regulated and down-regulated differentially accessible (DA) peaks for each main cell type were analyzed for transcription factor (TF) motif enrichment using HOMER (73). The scatter plot summarizes the number of cell types exhibiting significant motif enrichments (p.adj < 0.05) in either up-regulated (red) or down-regulated (blue) peak sets. TF motifs enriched across multiple cell types are labeled. **(B)** Dot plot showing the enrichment of interferon-stimulated response element (ISRE) motifs in aging-up DA peaks across cell types from diverse tissues. Dot size represents the fold change in ISRE-containing peaks in DA sets compared to a background peak set (**Methods**). Each dot represents one cell type from a specific tissue, colored by the tissue of origin. **(C)** Comparison of TF motif enrichment significance between female-specific and male-specific aging-up peak sets for each main cell type. Cell types with strong sex-specific motif enrichments are labeled. **(D)** Scatter plot showing enriched TF motifs in sex-shared, aging-up (red), and aging-down (blue) peaks from lung B cells. The x-axis denotes the difference in motif frequency (%) between DA and background peaks; the y-axis represents statistical significance. **(E)** Barplots showing motif enrichment for exemplary TFs: Ebf1 and Ets1 (down-regulated in both sexes), Irf2 (up-regulated in both sexes), and Pou2f2 (female-biased up-regulation) in lung B cells. **(F)** Scatterplot and barplot showing age-related expression changes of TFs highlighted in (**E**) in lung B cells, quantified using transcripts per million (TPM) for each animal. **(G)** Enrichment significance of EBF and IRF motifs for DA peaks from B cells across tissues, demonstrating conserved aging-associated regulatory patterns. **(H)** Scheme for computational analyses to identify cytokines potentially drive aging-associated chromatin remodeling in lung B cells. **(I)** Circle plot showing enrichments (−log10(p-value), hypergeometric test) of cytokine-specific signatures (derived from Immune Dictionary dataset (82)) in aging-associated genes identified in lung B cells from this study. **(J)** Dot plot displaying aggregated gene expressions of 350 genes shared between aging and cytokine treatments. Top conditions recapitulating aging-associated expression profiles were labeled. **(K)** Left: dot plot showing the enrichment score (−log10(p-value), hypergeometric test) of cytokine signatures in the male and female lung B cells as in **(I)**. Right: Heatmap indicating the aging-associated changes in the accessibility of cytokine secretion or receptor genes. **(L)** Genomic tracks showing aging-associated accessibility increases at *Ifng* and *Il21* in lung T cells and *Il4* and *Il2rb* in B cells. **(N)** Scatterplot showing increased gene expressions of *Il21* in T cells and *Il2rb* in B cells with age, together with a linear regression line. **(M)** Left, dot plot summarizing the enriched cytokine signatures using aging-up peak sets of B cells from each tissue. Only signatures that can be supported by increased accessibilities of secretion or receptor genes are shown. Right: barplot summarizing the number of tissues with activated signatures for each cytokine, splitting males and females.

We then explored upstream regulators of these aging-associated chromatin changes by performing motif enrichment analyses of DA peaks in each cell type. Aging-upregulated peaks were enriched for inflammatory motifs that are confirmed across multiple cell types, including IFN-stimulated response elements (ISRE) in 80 cell types, IRF1 in 77 cell types, IRF2 in 85 cell types, NF-κB in 57 cell types, and AP-1 in 71 cell types (Fig. 7A–B). In contrast, downregulated peaks were enriched for TF families linked to stem cell maintenance and development, such as *SOX15* ( in 18 cell types) and *E2A* (in 29 cell types) (Fig. 7A). Sex-specific patterns also emerged. For example, female-specific upregulated peaks were enriched for *POU* motifs in B cells and male-specific upregulated peaks were enriched for inflammatory TF motifs (IRFs and AP-1) in intestinal epithelial cells (Fig. 7C).

These TF activity changes were further supported by corresponding gene expression dynamics observed in single-cell RNA-seq data. For example, in the aged B cells in the lung, we detected reduced motif accessibility for Ebf1 and Ets1 (Fig. 7D–E), transcription factors involved in B cell development (25) and quiescent state maintenance (78), aligning with their decreased expression in both sexes (Fig. 7F). Meanwhile, aged B cells exhibited decreased expression and increased motif accessibility for Irf2 (Fig. 7D–E), reflecting its role as a transcriptional repressor of inflammatory signaling (79, 80). Pou2f2, a TF involved in B proliferation and plasma cell differentiation(81), showed increased motif accessibility exclusively in female-upregulated peak sets, corroborated by female-specific increases in Pou2f2 expression(Fig. 7D–E). This pattern is consistent with the preferential expansion of aging-associated B cells in female tissues (Fig. 4D and 4H). Moreover, the aging-associated changes in EBF and IRF motifs were detected in B cells body-wide, highlighting a global chromatin transition in B cells during aging (Fig. 7G).

Recognizing the strong inflammatory signature underlying these changes, we next explored whether cytokines known to drive immune responses could contribute to global epigenetic remodeling. Using the Immune Dictionary dataset (82), which encompasses scRNA-seq profiles of various immune cell types responding to 86 cytokines, we compared aging-related DA peaks and associated gene changes with those induced by different cytokines (Fig. 7H, **Methods**). This revealed that aging signatures, especially in female B cells from the lung, resembled those induced by specific cytokines such as interferons (IFNs), IL4, IL12, IL15, IL18, and IL21 (Fig. 7I). Cross-referencing aging-upregulated gene expression changes with cytokine-responsive transcripts further validated these links (Fig. 7J).

To reinforce this connection, we examined whether cytokine signatures were supported by changes in the accessibility of either secretion or receptor genes (Fig. 7K–L). For example, for secretion factors, we observed an increased accessibility of *Infg* and *Il21* in T cells, as well as *Il4* and *Il15* in B cells (Fig. 7L), aligning with activation of corresponding signatures, particularly in females (Fig. 7I). On the other hand, the upregulated response to IL2 and IL15 matched with increased accessibility of the receptor gene, *Il2rb*, in the aged B cells (Fig. 7L). A consistent trend was observed by analysis of recent snRNA-seq data (7), such as the increased expression of *Il21* in T cells, *Il2rb* and *Il15* in B cells (Fig. 7N and Fig. S10F).

Extending this analysis to all B cells across tissues uncovered the widespread emergence of cytokine signatures associated with aging, especially IL2, IL15, IFNγ, IL4, and IL21(Fig. 7M). Notably, the upregulated IL21 signatures were consistent with the global expansion of *Il21*+ T cells from in our previous analysis (T cells - 9, Fig. 4B and 4D) and were further confirmed at the plasma protein level in humans (3)(Fig. S10G). Interestingly, a previous study has described the function of IL21 in inducing the expression of Tbx21 (a marker of aging-associated B cells, Fig. 4B) in B cells and promoting their differentiation into plasma cells in systemic lupus erythematosus (83), suggesting a potential similar role of *Il21* in the epigenome reprogramming of B cells during aging. Similarly, we analyzed cytokine signatures in macrophages by leveraging expression changes following cytokine treatments of the same cell type from Immune Dictionary. This revealed activation of a distinct set of cytokines, including IL15, IL7, IL1α, IL18, and TNFα (Fig. S10H), that may underlie age-related molecular shifts in macrophages.

In summary, our integrative multi-level framework reveals a global, sex-dependent epigenetic reprogramming linked to inflammatory signaling and cytokine-driven pathways. These findings provide a roadmap for understanding the regulatory underpinnings of aging across diverse cell types and tissues—from intrinsic TF regulators to extrinsic cytokine signaling—and highlight candidate upstream factors that could be targeted to mitigate age-related epigenetic dysregulation.

## Discussion

Aging involves global deterioration in cellular and organismal function. While advancements in genomics techniques, especially single-cell RNA-seq and spatial transcriptomics, have been used to explore age-associated changes in cell-type- and region-specific gene expression (15, 57, 84, 85), few studies have systematically investigated cell population shifts, chromatin dynamics, as well as age-sex interaction on the scale of the entire organism. Here, by profiling around seven million cells from 21 mouse tissues at three ages and in both sexes, we constructed a single-cell chromatin accessibility atlas of aging with unprecedented depth and breadth. We reported the aging-associated dynamics of 536 tissue-level main cell types and 1,828 finer-grained subtypes, each defined by the accessibility at approximately 1.3 million cis-regulatory elements. Our findings reveal widespread cell population shifts, lineage-specific epigenomic reprogramming, and striking sex-dependent patterns, laying a foundation for understanding the regulatory logic of mammalian aging.

A key insight of our study is the extensive cell population dynamics accompanying age. Notably, 146 organ-specific cell types (approximately 25% of all organ-specific cell types) changed significantly with age. Many of these are immune cells. For example, we observed a widespread increase in plasma cells and macrophages across various tissues, aligning with previous findings of immunoglobulin accumulation (85) and monocyte/macrophage propagation (86) in aging. Tissue-specific population changes, such as the expansion of neutrophils in the liver and lung, and depletion of lymphoid progenitors in primary immune organs (e.g., pre-/pro-B cells in the bone marrow and immature T cells in the thymus) were identified. Beyond immune-related changes, our study also reveals additional vulnerabilities in non-immune cell lineages. For example, we observed profound depletion of specialized functional cell types across multiple organs, such as kidney podocytes, muscle tenocytes and satellite cells, ovary granulosa cells, and lung aerocytes. These depletions likely contribute to age-related loss of tissue homeostasis and functions.

Beyond shifts in broad cell types, our high-resolution analysis identified 1,828 subtypes—nearly one-third of which show significant aging dynamics. We identified subtypes exhibiting cell state changes that diverge from their parent population. For example, naive T cell subtypes in the lung and liver sharply decline, although total T cell proportions rise with age. Likewise, we detected region-specific vulnerability of endothelial cells in the kidney medulla and vein, distinct expansions in fibroblast and mast cell subpopulations in the intestine, and the emergence of a shared reactive state (*Creb5+ Bnc2+ Runx1+*) across different epithelial cell types in the kidney. These findings suggest that aging promotes a variety of cell-state transitions, including those localized to particular anatomical niches or widespread across distinct parent cell types potentially triggered by common circulating factors.

Coordinated expansion or depletion of the same subtype in multiple organs underscores a broader organism-level program. Immune subtypes, in particular, display strikingly synchronous changes, such as the global expansion of *Gzmk*+ CD8 T cells, regulatory T cells, *Il21*+ CD4 T cells, *Il23r*+ innate T cells and *Tbx21*+ B cells. Certain changes were restricted to a few organs, including the depletion of *Zfp683+* tissue-resident memory T cells in adipose and gut tissues, or the decline of *Gzma+* resting NK cells in spleen and bone marrow. However, this synchronization extends beyond mobile immune lineages: certain endothelial and fibroblast populations, also show parallel shifts across distinct tissues, such as the depletion of *Slc7a14*+ *Lhx6*+ *Meox2*+ vein endothelial cell and the expansion of an *Ifi207+ Ear1+* aging-associated fibroblast subtype. This points to systemic inflammatory signals or hormonal cues that modulate cell fates body-wide.

At the molecular level, we identified extensive chromatin reprogramming associated with aging. Our differential accessibility analyses uncovered 279,401 peaks changing with age, including a subset consistently altered across many cell types (e.g., promoter peaks of *Nr3c2* and *Il7* that frequently gain accessibility, and *Sox4* or *Sox11* peaks that frequently lose accessibility). Linking chromatin changes to RNA-seq data validated the effects from accessibilities to expressions and revealed putative enhancers regulating aging-associated genes. Motif analysis further supports a shift from TFs involved in tissue maintenance or stemness (*e.g., SOX, E2A*) to those mediating inflammatory and stress responses (*e.g., NF-κB, IRFs, AP-1*). Specifically, as an example to showcase the potential of our data to reveal anti-aging targets, we focused on cytokines and identified the activation of IL15, IL21, IL4, and IFNγ signatures in aged B cells, indicating these extrinsic signals may reinforce or drive the cell-state transitions observed in aging.

Crucially, we found extensive sexual dimorphism. Around forty percent of aging-associated main cell types (55 out of 146) and subtypes (193 out of 499) exhibited sex differences. Notable examples include the male-biased expansion of hepatic stellate cells in the liver and the female-biased expansion of interstitial macrophages in the lung. Overall, female mice appeared to mount a more pronounced immune activation in aging at both proportional and molecular levels, consistent with the higher incidence of autoimmune diseases in women (87, 88). This included female-specific upregulation of TFs like *Pou2f2* in B cells, aligning with female-biased expansions of aging-associated B cell subtypes. We also observed subtypes that shift in opposite directions by sex, such as *Slc22a7*+ proximal tubule cells (PT S3T2) increasing in aged females but decreasing in males—trends replicated by single-cell transcriptomic data. At the epigenetic level, we detected tens of thousands of cell-type-specific peaks that changed exclusively in one sex, highlighting sex as a major axis of aging heterogeneity.

In summary, this organism-level single-cell chromatin accessibility atlas illuminates how aging remodels the cellular composition and regulatory landscape of multiple tissues. Although many changes are highly tissue-specific, we uncovered “coordinated” cellular and molecular dynamics that are shared across different organs, including immune remodeling, broad depletion of functional cell types, the emergence of inflammation-related states, and sex-dependent trajectories. By cataloging these changes, we offer a resource for understanding the molecular logic of aging and for guiding therapeutic strategies aimed at preserving or restoring youthful tissue states. This work also highlights molecular candidates to mitigate aging-related dysfunction by targeting broad-spectral cell types across tissues, including intrinsic transcription factors, extrinsic cytokines, and individual receptors (*Nr3c2* for immune cells, *Epha3* for T cells, *Il2rb* for B cells). Looking forward, we anticipate that this atlas will serve as a critical reference for evaluating anti-aging interventions and refining our understanding of how chromatin remodeling, cell state transitions, and tissue physiology intersect in the aging mammalian body.

## Supplementary Material

Supplement 1

Supplement 2

## Data Availability

Raw FASTQ files, processed count matrices, cell metadata, and peak metadata can be downloaded from NCBI GEO under accession number GSE288730 (reviewer’s token: exitcckgbfmzpkl). An interactive website facilitating the visualization of our data across cell type, age, and sex is available at https://mouseagingatacatlas.org.

## References

[1] Niccoli T., Partridge L., Ageing as a risk factor for disease. Curr Biol 22, R741–52 (2012). 22975005 10.1016/j.cub.2012.07.024

[2] Tian Y. E., Cropley V., Maier A. B., Lautenschlager N. T., Breakspear M., Zalesky A., Heterogeneous aging across multiple organ systems and prediction of chronic disease and mortality. Nat Med 29, 1221–1231 (2023). 37024597 10.1038/s41591-023-02296-6

[3] Oh H. S.-H., Rutledge J., Nachun D., Pálovics R., Abiose O., Moran-Losada P., Channappa D., Urey D. Y., Kim K., Sung Y. J., Wang L., Timsina J., Western D., Liu M., Kohlfeld P., Budde J., Wilson E. N., Guen Y., Maurer T. M., Haney M., Yang A. C., He Z., Greicius M. D., Andreasson K. I., Sathyan S., Weiss E. F., Milman S., Barzilai N., Cruchaga C., Wagner A. D., Mormino E., Lehallier B., Henderson V. W., Longo F. M., Montgomery S. B., Wyss-Coray T., Organ aging signatures in the plasma proteome track health and disease. Nature 624, 164–172 (2023). 38057571 10.1038/s41586-023-06802-1 PMC10700136

[4] Jin K., Yao Z., van Velthoven C. T. J., Kaplan E. S., Glattfelder K., Barlow S. T., Boyer G., Carey D., Casper T., Chakka A. B., Chakrabarty R., Clark M., Departee M., Desierto M., Gary A., Gloe J., Goldy J., Guilford N., Guzman J., Hirschstein D., Lee C., Liang E., Pham T., Reding M., Ronellenfitch K., Ruiz A., Sevigny J., Shapovalova N., Shulga L., Sulc J., Torkelson A., Tung H., Levi B., Sunkin S. M., Dee N., Esposito L., Smith K. A., Tasic B., Zeng H., Brain-wide cell-type-specific transcriptomic signatures of healthy ageing in mice. Nature, doi: 10.1038/s41586-024-08350-8 (2025). PMC11798837 39743592

[5] Wang R., Zhang P., Wang J., Ma L., W. E, Suo S., Jiang M., Li J., Chen H., Sun H., Fei L., Zhou Z., Zhou Y., Chen Y., Zhang W., Wang X., Mei Y., Sun Z., Yu C., Shao J., Fu Y., Xiao Y., Ye F., Fang X., Wu H., Guo Q., Fang X., Li X., Gao X., Wang D., Xu P.-F., Zeng R., Xu G., Zhu L., Wang L., Qu J., Zhang D., Ouyang H., Huang H., Chen M., Ng S.-C., Liu G.-H., Yuan G.-C., Guo G., Han X., Construction of a cross-species cell landscape at single-cell level. Nucleic Acids Res 51, 501–516 (2023). 35929025 10.1093/nar/gkac633 PMC9881150

[6] Lu T.-C., Brbić M., Park Y.-J., Jackson T., Chen J., Kolluru S. S., Qi Y., Katheder N. S., Cai X. T., Lee S., Chen Y.-C., Auld N., Liang C.-Y., Ding S. H., Welsch D., D’Souza S., Pisco A. O., Jones R. C., Leskovec J., Lai E. C., Bellen H. J., Luo L., Jasper H., Quake S. R., Li H., Aging Fly Cell Atlas identifies exhaustive aging features at cellular resolution. Science 380, eadg0934 (2023). 37319212 10.1126/science.adg0934 PMC10829769

[7] Zhang Z., Schaefer C., Jiang W., Lu Z., Lee J., Sziraki A., Abdulraouf A., Wick B., Haeussler M., Li Z., Molla G., Satija R., Zhou W., Cao J., A panoramic view of cell population dynamics in mammalian aging. Science 387, eadn3949 (2025). 39607904 10.1126/science.adn3949 PMC11910726

[8] Beintema J. J., Campagne R. N., Gruber M., Rat pancreatic ribonuclease. I. Isolation and properties. Biochim Biophys Acta 310, 148–160 (1973). 4710591 10.1016/0005-2795(73)90019-6

[9] Al-Adsani A. M., Barhoush S. A., Bastaki N. K., Al-Bustan S. A., Al-Qattan K. K., Comparing and Optimizing RNA Extraction from the Pancreas of Diabetic and Healthy Rats for Gene Expression Analyses. Genes (Basel) 13 (2022). 10.3390/genes13050881 PMC9141713 35627265

[10] De Rop F. V., Hulselmans G., Flerin C., Soler-Vila P., Rafels A., Christiaens V., González-Blas C. B., Marchese D., Caratù G., Poovathingal S., Rozenblatt-Rosen O., Slyper M., Luo W., Muus C., Duarte F., Shrestha R., Bagdatli S. T., Corces M. R., Mamanova L., Knights A., Meyer K. B., Mulqueen R., Taherinasab A., Maschmeyer P., Pezoldt J., Lambert C. L. G., Iglesias M., Najle S. R., Dossani Z. Y., Martelotto L. G., Burkett Z., Lebofsky R., Martin-Subero J. I., Pillai S., Sebé-Pedrós A., Deplancke B., Teichmann S. A., Ludwig L. S., Braun T. P., Adey A. C., Greenleaf W. J., Buenrostro J. D., Regev A., Aerts S., Heyn H., Systematic benchmarking of single-cell ATAC-sequencing protocols. Nat Biotechnol 42, 916–926 (2024). 37537502 10.1038/s41587-023-01881-x PMC11180611

[11] Domcke S., Hill A. J., Daza R. M., Cao J., O’Day D. R., Pliner H. A., Aldinger K. A., Pokholok D., Zhang F., Milbank J. H., Zager M. A., Glass I. A., Steemers F. J., Doherty D., Trapnell C., Cusanovich D. A., Shendure J., A human cell atlas of fetal chromatin accessibility. Science 370 (2020). 10.1126/science.aba7612 PMC7785298 33184180

[12] Zhang K., Hocker J. D., Miller M., Hou X., Chiou J., Poirion O. B., Qiu Y., Li Y. E., Gaulton K. J., Wang A., Preissl S., Ren B., A single-cell atlas of chromatin accessibility in the human genome. Cell 184, 5985–6001.e19 (2021). 34774128 10.1016/j.cell.2021.10.024 PMC8664161

[13] Li Y. E., Preissl S., Miller M., Johnson N. D., Wang Z., Jiao H., Zhu C., Wang Z., Xie Y., Poirion O., Kern C., Pinto-Duarte A., Tian W., Siletti K., Emerson N., Osteen J., Lucero J., Lin L., Yang Q., Zhu Q., Zemke N., Espinoza S., Yanny A. M., Nyhus J., Dee N., Casper T., Shapovalova N., Hirschstein D., Hodge R. D., Linnarsson S., Bakken T., Levi B., Keene C. D., Shang J., Lein E., Wang A., Behrens M. M., Ecker J. R., Ren B., A comparative atlas of single-cell chromatin accessibility in the human brain. Science 382, eadf7044 (2023). 37824643 10.1126/science.adf7044 PMC10852054

[14] Zu S., Li Y. E., Wang K., Armand E. J., Mamde S., Amaral M. L., Wang Y., Chu A., Xie Y., Miller M., Xu J., Wang Z., Zhang K., Jia B., Hou X., Lin L., Yang Q., Lee S., Li B., Kuan S., Liu H., Zhou J., Pinto-Duarte A., Lucero J., Osteen J., Nunn M., Smith K. A., Tasic B., Yao Z., Zeng H., Wang Z., Shang J., Behrens M. M., Ecker J. R., Wang A., Preissl S., Ren B., Single-cell analysis of chromatin accessibility in the adult mouse brain. Nature 624, 378–389 (2023). 38092917 10.1038/s41586-023-06824-9 PMC10719105

[15] Sziraki A., Lu Z., Lee J., Banyai G., Anderson S., Abdulraouf A., Metzner E., Liao A., Banfelder J., Epstein A., Schaefer C., Xu Z., Zhang Z., Gan L., Nelson P. T., Zhou W., Cao J., A global view of aging and Alzheimer’s pathogenesis-associated cell population dynamics and molecular signatures in human and mouse brains. Nature Genetics 55, 2104–2116 (2023). 38036784 10.1038/s41588-023-01572-y PMC10703679

[16] Lu Z., Zhang M., Lee J., Sziraki A., Anderson S., Zhang Z., Xu Z., Jiang W., Ge S., Nelson P. T., Zhou W., Cao J., Tracking cell-type-specific temporal dynamics in human and mouse brains. Cell 186, 4345–4364.e24 (2023). 37774676 10.1016/j.cell.2023.08.042 PMC10545416

[17] Zhang K., Zemke N. R., Armand E. J., Ren B., A fast, scalable and versatile tool for analysis of single-cell omics data. Nature Methods 21, 217–227 (2024). 38191932 10.1038/s41592-023-02139-9 PMC10864184

[18] Charrier A., Brigstock D. R., Regulation of pancreatic function by connective tissue growth factor (CTGF, CCN2). Cytokine Growth Factor Rev 24, 59–68 (2013). 22884427 10.1016/j.cytogfr.2012.07.001 PMC3508350

[19] Segerstolpe Å., Palasantza A., Eliasson P., Andersson E.-M., Andréasson A.-C., Sun X., Picelli S., Sabirsh A., Clausen M., Bjursell M. K., Smith D. M., Kasper M., Ämmälä C., Sandberg R., Single-Cell Transcriptome Profiling of Human Pancreatic Islets in Health and Type 2 Diabetes. Cell Metab 24, 593–607 (2016). 27667667 10.1016/j.cmet.2016.08.020 PMC5069352

[20] Moore J. E., Purcaro M. J., Pratt H. E., Epstein C. B., Shoresh N., Adrian J., Kawli T., Davis C. A., Dobin A., Kaul R., Halow J., Van Nostrand E. L., Freese P., Gorkin D. U., Shen Y., He Y., Mackiewicz M., Pauli-Behn F., Williams B. A., Mortazavi A., Keller C. A., Zhang X.-O., Elhajjajy S. I., Huey J., Dickel D. E., Snetkova V., Wei X., Wang X., Rivera-Mulia J. C., Rozowsky J., Zhang J., Chhetri S. B., Zhang J., Victorsen A., White K. P., Visel A., Yeo G. W., Burge C. B., Lécuyer E., Gilbert D. M., Dekker J., Rinn J., Mendenhall E. M., Ecker J. R., Kellis M., Klein R. J., Noble W. S., Kundaje A., Guigó R., Farnham P. J., Cherry J. M., Myers R. M., Ren B., Graveley B. R., Gerstein M. B., Pennacchio L. A., Snyder M. P., Bernstein B. E., Wold B., Hardison R. C., Gingeras T. R., Stamatoyannopoulos J. A., Weng Z., Expanded encyclopaedias of DNA elements in the human and mouse genomes. Nature 583, 699–710 (2020). 32728249 10.1038/s41586-020-2493-4 PMC7410828

[21] Schep A. N., Wu B., Buenrostro J. D., Greenleaf W. J., chromVAR: inferring transcription-factor-associated accessibility from single-cell epigenomic data. Nat. Methods 14, 975–978 (2017). 28825706 10.1038/nmeth.4401 PMC5623146

[22] Shan Q., Zeng Z., Xing S., Li F., Hartwig S. M., Gullicksrud J. A., Kurup S. P., Van Braeckel-Budimir N., Su Y., Martin M. D., Varga S. M., Taniuchi I., Harty J. T., Peng W., Badovinac V. P., Xue H.-H., The transcription factor Runx3 guards cytotoxic CD8 effector T cells against deviation towards follicular helper T cell lineage. Nat Immunol 18, 931–939 (2017). 28604718 10.1038/ni.3773 PMC5564218

[23] Laiosa C. V., Stadtfeld M., Graf T., Determinants of lymphoid-myeloid lineage diversification. Annu Rev Immunol 24, 705–738 (2006). 16551264 10.1146/annurev.immunol.24.021605.090742

[24] Klein U., Casola S., Cattoretti G., Shen Q., Lia M., Mo T., Ludwig T., Rajewsky K., Dalla-Favera R., Transcription factor IRF4 controls plasma cell differentiation and class-switch recombination. Nat Immunol 7, 773–782 (2006). 16767092 10.1038/ni1357

[25] Nechanitzky R., Akbas D., Scherer S., Györy I., Hoyler T., Ramamoorthy S., Diefenbach A., Grosschedl R., Transcription factor EBF1 is essential for the maintenance of B cell identity and prevention of alternative fates in committed cells. Nature Immunology 14, 867–875 (2013). 23812095 10.1038/ni.2641

[26] Pagani F., Tratta E., Dell’Era P., Cominelli M., Poliani P. L., EBF1 is expressed in pericytes and contributes to pericyte cell commitment. Histochem Cell Biol 156, 333–347 (2021). 34272603 10.1007/s00418-021-02015-7 PMC8550016

[27] McClellan D., Casey M. J., Bareyan D., Lucente H., Ours C., Velinder M., Singer J., Lone M. D., Sun W., Coria Y., Mason C. C., Engel M. E., Growth Factor Independence 1B-Mediated Transcriptional Repression and Lineage Allocation Require Lysine-Specific Demethylase 1-Dependent Recruitment of the BHC Complex. Mol Cell Biol 39 (2019). 10.1128/MCB.00020-19 PMC6580704 30988160

[28] Bulik-Sullivan B. K., Loh P.-R., Finucane H. K., Ripke S., Yang J., Patterson N., Daly M. J., Price A. L., Neale B. M., LD Score regression distinguishes confounding from polygenicity in genome-wide association studies. Nature Genetics 47, 291–295 (2015). 25642630 10.1038/ng.3211 PMC4495769

[29] Li Y., Laws S. M., Miles L. A., Wiley J. S., Huang X., Masters C. L., Gu B. J., Genomics of Alzheimer’s disease implicates the innate and adaptive immune systems. Cell Mol Life Sci 78, 7397–7426 (2021). 34708251 10.1007/s00018-021-03986-5 PMC11073066

[30] Scholz C., Patton K. T., Anderson D. E., Freeman G. J., Hafler D. A., Expansion of autoreactive T cells in multiple sclerosis is independent of exogenous B7 costimulation. J Immunol 160, 1532–1538 (1998). 9570577

[31] Comi G., Bar-Or A., Lassmann H., Uccelli A., Hartung H.-P., Montalban X., Sørensen P. S., Hohlfeld R., Hauser S. L., Expert Panel of the 27th Annual Meeting of the European Charcot Foundation, Role of B Cells in Multiple Sclerosis and Related Disorders. Ann Neurol 89, 13–23 (2021). 33091175 10.1002/ana.25927 PMC8007167

[32] Goldstein J. L., Brown M. S., Regulation of low-density lipoprotein receptors: implications for pathogenesis and therapy of hypercholesterolemia and atherosclerosis. Circulation 76, 504–507 (1987). 3621516 10.1161/01.cir.76.3.504

[33] Wysham C., Shubrook J., Beta-cell failure in type 2 diabetes: mechanisms, markers, and clinical implications. Postgrad Med 132, 676–686 (2020). 32543261 10.1080/00325481.2020.1771047

[34] Yamaguchi H., Gomez R. A., Sequeira-Lopez M. L. S., Renin Cells, From Vascular Development to Blood Pressure Sensing. Hypertension 80, 1580–1589 (2023). 37313725 10.1161/HYPERTENSIONAHA.123.20577 PMC10526986

[35] Rodríguez-Montes L., Ovchinnikova S., Yuan X., Studer T., Sarropoulos I., Anders S., Kaessmann H., Cardoso-Moreira M., Sex-biased gene expression across mammalian organ development and evolution. Science, doi: 10.1126/science.adf1046 (2023). PMC7615307 37917687

[36] Christianto A., Baba T., Takahashi F., Inui K., Inoue M., Suyama M., Ono Y., Ohkawa Y., Morohashi K.-I., Sex differences in metabolic pathways are regulated by Pfkfb3 and Pdk4 expression in rodent muscle. Commun Biol 4, 1264 (2021). 34737380 10.1038/s42003-021-02790-y PMC8569015

[37] Janosevic D., Myslinski J., McCarthy T. W., Zollman A., Syed F., Xuei X., Gao H., Liu Y.-L., Collins K. S., Cheng Y.-H., Winfree S., El-Achkar T. M., Maier B., Melo Ferreira R., Eadon M. T., Hato T., Dagher P. C., The orchestrated cellular and molecular responses of the kidney to endotoxin define a precise sepsis timeline. Elife 10 (2021). 10.7554/eLife.62270 PMC7810465 33448928

[38] Jagger A., Shimojima Y., Goronzy J. J., Weyand C. M., Regulatory T cells and the immune aging process: a mini-review. Gerontology 60, 130–137 (2014). 24296590 10.1159/000355303 PMC4878402

[39] Li X., Li C., Zhang W., Wang Y., Qian P., Huang H., Inflammation and aging: signaling pathways and intervention therapies. Signal Transduct Target Ther 8, 239 (2023). 37291105 10.1038/s41392-023-01502-8 PMC10248351

[40] Frasca D., Romero M., Garcia D., Diaz A., Blomberg B. B., Hyper-metabolic B cells in the spleens of old mice make antibodies with autoimmune specificities. Immun Ageing 18, 9 (2021). 33639971 10.1186/s12979-021-00222-3 PMC7916295

[41] Hilmer S. N., Cogger V. C., Le Couteur D. G., Basal activity of Kupffer cells increases with old age. J Gerontol A Biol Sci Med Sci 62, 973–978 (2007). 17895435 10.1093/gerona/62.9.973

[42] Mukherjee S., Bruno M. E. C., Oakes J., Hawk G. S., Stromberg A. J., Cohen D. A., Starr M. E., Mechanisms of γδ T cell accumulation in visceral adipose tissue with aging. Front Aging 4, 1258836 (2023). 38274288 10.3389/fragi.2023.1258836 PMC10808514

[43] Systematic analysis of muscle aging using joint single-cell and single-nucleus sequencing. Nat Aging 4, 621–622 (2024). 38658781 10.1038/s43587-024-00629-9

[44] Gribble F. M., Reimann F., Function and mechanisms of enteroendocrine cells and gut hormones in metabolism. Nat Rev Endocrinol 15, 226–237 (2019). 30760847 10.1038/s41574-019-0168-8

[45] de Mol J., Kuiper J., Tsiantoulas D., Foks A. C., The Dynamics of B Cell Aging in Health and Disease. Front Immunol 12, 733566 (2021). 34675924 10.3389/fimmu.2021.733566 PMC8524000

[46] Aspinall R., Age-associated thymic atrophy in the mouse is due to a deficiency affecting rearrangement of the TCR during intrathymic T cell development. J Immunol 158, 3037–3045 (1997). 9120255

[47] Menees K. B., Earls R. H., Chung J., Jernigan J., Filipov N. M., Carpenter J. M., Lee J.-K., Sex- and age-dependent alterations of splenic immune cell profile and NK cell phenotypes and function in C57BL/6J mice. Immun Ageing 18, 3 (2021). 33419446 10.1186/s12979-021-00214-3 PMC7791703

[48] Zacca E. R., Crespo M. I., Acland R. P., Roselli E., Núñez N. G., Maccioni M., Maletto B. A., Pistoresi-Palencia M. C., Morón G., Aging Impairs the Ability of Conventional Dendritic Cells to Cross-Prime CD8+ T Cells upon Stimulation with a TLR7 Ligand. PLoS One 10, e0140672 (2015). 26474053 10.1371/journal.pone.0140672 PMC4608578

[49] Jin C., Wang X., Yang J., Kim S., Hudgins A. D., Gamliel A., Pei M., Contreras D., Devos M., Guo Q., Vijg J., Conti M., Hoeijmakers J., Campisi J., Lobo R., Williams Z., Rosenfeld M. G., Suh Y., Molecular and genetic insights into human ovarian aging from single-nuclei multi-omics analyses. Nat Aging, doi: 10.1038/s43587-024-00762-5 (2024). PMC11839473 39578560

[50] Broekmans F. J., Soules M. R., Fauser B. C., Ovarian aging: mechanisms and clinical consequences. Endocr Rev 30, 465–493 (2009). 19589949 10.1210/er.2009-0006

[51] Ortonne J. P., Pigmentary changes of the ageing skin. Br J Dermatol 122 Suppl 35, 21–28 (1990). 10.1111/j.1365-2133.1990.tb16121.x 2186781

[52] Kataru R. P., Park H. J., Shin J., Baik J. E., Sarker A., Brown S., Mehrara B. J., Structural and Functional Changes in Aged Skin Lymphatic Vessels. Front Aging 3, 864860 (2022). 35821848 10.3389/fragi.2022.864860 PMC9261401

[53] Gillich A., Zhang F., Farmer C. G., Travaglini K. J., Tan S. Y., Gu M., Zhou B., Feinstein J. A., Krasnow M. A., Metzger R. J., Capillary cell-type specialization in the alveolus. Nature 586, 785–789 (2020). 33057196 10.1038/s41586-020-2822-7 PMC7721049

[54] Hermann E. A., Motahari A., Hoffman E. A., Allen N., Bertoni A. G., Bluemke D. A., Eskandari A., Gerard S. E., Guo J., Hiura G. T., Kaczka D. W., Michos E. D., Nagpal P., Pankow J., Shah S., Smith B. M., Hinckley Stukovsky K., Sun Y., Watson K., Barr R. G., Pulmonary Blood Volume Among Older Adults in the Community: The MESA Lung Study. Circ Cardiovasc Imaging 15, e014380 (2022). 35938411 10.1161/CIRCIMAGING.122.014380 PMC9387743

[55] Shankland S. J., Wang Y., Shaw A. S., Vaughan J. C., Pippin J. W., Wessely O., Podocyte Aging: Why and How Getting Old Matters. J Am Soc Nephrol 32, 2697–2713 (2021). 34716239 10.1681/ASN.2021050614 PMC8806106

[56] Liu W.-B., Huang G.-R., Liu B.-L., Hu H.-K., Geng J., Rui H.-L., Gao C., Huang Y.-J., Huo G.-Y., Mao J.-R., Lu C.-J., Xu A.-L., Single cell landscape of parietal epithelial cells in healthy and diseased states. Kidney Int 104, 108–123 (2023). 37100348 10.1016/j.kint.2023.03.036

[57] Tabula Muris Consortium, A single-cell transcriptomic atlas characterizes ageing tissues in the mouse. Nature 583, 590–595 (2020). 32669714 10.1038/s41586-020-2496-1 PMC8240505

[58] Tsai W.-C., Chang H.-N., Yu T.-Y., Chien C.-H., Fu L.-F., Liang F.-C., Pang J.-H. S., Decreased proliferation of aging tenocytes is associated with down-regulation of cellular senescence-inhibited gene and up-regulation of p27. J Orthop Res 29, 1598–1603 (2011). 21452304 10.1002/jor.21418

[59] Chakkalakal J. V., Jones K. M., Basson M. A., Brack A. S., The aged niche disrupts muscle stem cell quiescence. Nature 490, 355–360 (2012). 23023126 10.1038/nature11438 PMC3605795

[60] Pellegrino R., Paganelli R., Di Iorio A., Bandinelli S., Moretti A., Iolascon G., Sparvieri E., Tarantino D., Ferrucci L., Temporal trends, sex differences, and age-related disease influence in Neutrophil, Lymphocyte count and Neutrophil to Lymphocyte-ratio: results from InCHIANTI follow-up study. Immun Ageing 20, 46 (2023). 37667259 10.1186/s12979-023-00370-8 PMC10476368

[61] Busse P. J., Mathur S. K., Age-related changes in immune function: effect on airway inflammation. J Allergy Clin Immunol 126, 690–9; quiz 700–1 (2010). 20920759 10.1016/j.jaci.2010.08.011 PMC3297963

[62] Li J., Choi J., Cheng X., Ma J., Pema S., Sanes J. R., Mardon G., Frankfort B. J., Tran N. M., Li Y., Chen R., Comprehensive single-cell atlas of the mouse retina. iScience 27, 109916 (2024). 38812536 10.1016/j.isci.2024.109916 PMC11134544

[63] Purohit S. J., Stephan R. P., Kim H.-G., Herrin B. R., Gartland L., Klug C. A., Determination of lymphoid cell fate is dependent on the expression status of the IL-7 receptor. EMBO J 22, 5511–5521 (2003). 14532123 10.1093/emboj/cdg522 PMC213776

[64] Poscablo D. M., Worthington A. K., Smith-Berdan S., Rommel M. G. E., Manso B. A., Adili R., Mok L., Reggiardo R. E., Cool T., Mogharrab R., Myers J., Dahmen S., Medina P., Beaudin A. E., Boyer S. W., Holinstat M., Jonsson V. D., Forsberg E. C., An age-progressive platelet differentiation path from hematopoietic stem cells causes exacerbated thrombosis. Cell 187, 3090–3107.e21 (2024). 38749423 10.1016/j.cell.2024.04.018 PMC12047039

[65] Dumas S. J., Meta E., Borri M., Goveia J., Rohlenova K., Conchinha N. V., Falkenberg K., Teuwen L.-A., de Rooij L., Kalucka J., Chen R., Khan S., Taverna F., Lu W., Parys M., De Legher C., Vinckier S., Karakach T. K., Schoonjans L., Lin L., Bolund L., Dewerchin M., Eelen G., Rabelink T. J., Li X., Luo Y., Carmeliet P., Single-Cell RNA Sequencing Reveals Renal Endothelium Heterogeneity and Metabolic Adaptation to Water Deprivation. J Am Soc Nephrol 31, 118–138 (2020). 31818909 10.1681/ASN.2019080832 PMC6935008

[66] Mogilenko D. A., Shchukina I., Artyomov M. N., Immune ageing at single-cell resolution. Nature Reviews Immunology 22, 484–498 (2021). 10.1038/s41577-021-00646-4 PMC8609266 34815556

[67] Kohyama M., Ise W., Edelson B. T., Wilker P. R., Hildner K., Mejia C., Frazier W. A., Murphy T. L., Murphy K. M., Role for Spi-C in the development of red pulp macrophages and splenic iron homeostasis. Nature 457, 318–321 (2009). 19037245 10.1038/nature07472 PMC2756102

[68] Fehniger T. A., Cai S. F., Cao X., Bredemeyer A. J., Presti R. M., French A. R., Ley T. J., Acquisition of murine NK cell cytotoxicity requires the translation of a pre-existing pool of granzyme B and perforin mRNAs. Immunity 26, 798–811 (2007). 17540585 10.1016/j.immuni.2007.04.010

[69] Chiurchiù V., Lanuti M., De Bardi M., Battistini L., Maccarrone M., The differential characterization of GPR55 receptor in human peripheral blood reveals a distinctive expression in monocytes and NK cells and a proinflammatory role in these innate cells. Int Immunol 27, 153–160 (2015). 25344934 10.1093/intimm/dxu097

[70] Gender differences in autoimmune disease. Frontiers in Neuroendocrinology 35, 347–369 (2014). 24793874 10.1016/j.yfrne.2014.04.004

[71] Elderman M., de Vos P., Faas M., Role of Microbiota in Sexually Dimorphic Immunity. Front Immunol 9, 1018 (2018). 29910797 10.3389/fimmu.2018.01018 PMC5992421

[72] Kalucka J., de Rooij L. P. M. H., Goveia J., Rohlenova K., Dumas S. J., Meta E., Conchinha N. V., Taverna F., Teuwen L.-A., Veys K., García-Caballero M., Khan S., Geldhof V., Sokol L., Chen R., Treps L., Borri M., de Zeeuw P., Dubois C., Karakach T. K., Falkenberg K. D., Parys M., Yin X., Vinckier S., Du Y., Fenton R. A., Schoonjans L., Dewerchin M., Eelen G., Thienpont B., Lin L., Bolund L., Li X., Luo Y., Carmeliet P., Single-Cell Transcriptome Atlas of Murine Endothelial Cells. Cell 180, 764–779.e20 (2020). 32059779 10.1016/j.cell.2020.01.015

[73] Heinz S., Benner C., Spann N., Bertolino E., Lin Y. C., Laslo P., Cheng J. X., Murre C., Singh H., Glass C. K., Simple combinations of lineage-determining transcription factors prime cis-regulatory elements required for macrophage and B cell identities. Mol Cell 38, 576–589 (2010). 20513432 10.1016/j.molcel.2010.05.004 PMC2898526

[74] Palomero J., Vegliante M. C., Rodríguez M. L., Eguileor A., Castellano G., Planas-Rigol E., Jares P., Ribera-Cortada I., Cid M. C., Campo E., Amador V., SOX11 promotes tumor angiogenesis through transcriptional regulation of PDGFA in mantle cell lymphoma. Blood 124, 2235–2247 (2014). 25092176 10.1182/blood-2014-04-569566

[75] Orang A., Dredge B. K., Liu C. Y., Bracken J. M., Chen C.-H., Sourdin L., Whitfield H. J., Lumb R., Boyle S. T., Davis M. J., Samuel M. S., Gregory P. A., Khew-Goodall Y., Goodall G. J., Pillman K. A., Bracken C. P., Basonuclin-2 regulates extracellular matrix production and degradation. Life Sci Alliance 6 (2023). 10.26508/lsa.202301984 PMC10400885 37536977

[76] Li Y., Zhang J., Zhai P., Hu C., Suo J., Wang J., Liu C., Peng Z., The potential biomarker TIFA regulates pyroptosis in sepsis-induced acute kidney injury. Int Immunopharmacol 115, 109580 (2023). 36586274 10.1016/j.intimp.2022.109580

[77] Robinson M. D., McCarthy D. J., Smyth G. K., edgeR: a Bioconductor package for differential expression analysis of digital gene expression data. Bioinformatics 26, 139–140 (2010). 19910308 10.1093/bioinformatics/btp616 PMC2796818

[78] John S. A., Clements J. L., Russell L. M., Garrett-Sinha L. A., Ets-1 regulates plasma cell differentiation by interfering with the activity of the transcription factor Blimp-1. J Biol Chem 283, 951–962 (2008). 17977828 10.1074/jbc.M705262200

[79] Yamamoto H., Lamphier M. S., Fujita T., Taniguchi T., Harada H., The oncogenic transcription factor IRF-2 possesses a transcriptional repression and a latent activation domain. Oncogene 9, 1423–1428 (1994). 8152803

[80] Cui H., Banerjee S., Guo S., Xie N., Liu G., IFN Regulatory Factor 2 Inhibits Expression of Glycolytic Genes and Lipopolysaccharide-Induced Proinflammatory Responses in Macrophages. J Immunol 200, 3218–3230 (2018). 29563175 10.4049/jimmunol.1701571 PMC5915871

[81] Hodson D. J., Shaffer A. L., Xiao W., Wright G. W., Schmitz R., Phelan J. D., Yang Y., Webster D. E., Rui L., Kohlhammer H., Nakagawa M., Waldmann T. A., Staudt L. M., Regulation of normal B-cell differentiation and malignant B-cell survival by OCT2. Proc Natl Acad Sci U S A 113, E2039–46 (2016). 26993806 10.1073/pnas.1600557113 PMC4833274

[82] Cui A., Huang T., Li S., Ma A., Pérez J. L., Sander C., Keskin D. B., Wu C. J., Fraenkel E., Hacohen N., Dictionary of immune responses to cytokines at single-cell resolution. Nature 625, 377–384 (2024). 38057668 10.1038/s41586-023-06816-9 PMC10781646

[83] Wang S., Wang J., Kumar V., Karnell J. L., Naiman B., Gross P. S., Rahman S., Zerrouki K., Hanna R., Morehouse C., Holoweckyj N., Liu H., Manna Z., Goldbach-Mansky R., Hasni S., Siegel R., Sanjuan M., Streicher K., Cancro M. P., Kolbeck R., Ettinger R., IL-21 drives expansion and plasma cell differentiation of autoreactive CD11chiT-bet+ B cells in SLE. Nature Communications 9, 1–14 (2018). 10.1038/s41467-018-03750-7 PMC5931508 29717110

[84] Terekhova M., Swain A., Bohacova P., Aladyeva E., Arthur L., Laha A., Mogilenko D. A., Burdess S., Sukhov V., Kleverov D., Echalar B., Tsurinov P., Chernyatchik R., Husarcikova K., Artyomov M. N., Single-cell atlas of healthy human blood unveils age-related loss of NKG2CGZMBCD8 memory T cells and accumulation of type 2 memory T cells. Immunity 56, 2836–2854.e9 (2023). 37963457 10.1016/j.immuni.2023.10.013

[85] Ma S., Ji Z., Zhang B., Geng L., Cai Y., Nie C., Li J., Zuo Y., Sun Y., Xu G., Liu B., Ai J., Liu F., Zhao L., Zhang J., Zhang H., Sun S., Huang H., Zhang Y., Ye Y., Fan Y., Zheng F., Hu J., Zhang B., Li J., Feng X., Zhang F., Zhuang Y., Li T., Yu Y., Bao Z., Pan S., Rodriguez Esteban C., Liu Z., Deng H., Wen F., Song M., Wang S., Zhu G., Yang J., Jiang T., Song W., Izpisua Belmonte J. C., Qu J., Zhang W., Gu Y., Liu G.-H., Spatial transcriptomic landscape unveils immunoglobin-associated senescence as a hallmark of aging. Cell 187, 7025–7044.e34 (2024). 39500323 10.1016/j.cell.2024.10.019

[86] Duong L., Pixley F. J., Nelson D. J., Jackaman C., Aging Leads to Increased Monocytes and Macrophages With Altered CSF-1 Receptor Expression and Earlier Tumor-Associated Macrophage Expansion in Murine Mesothelioma. Front Aging 3, 848925 (2022). 35821822 10.3389/fragi.2022.848925 PMC9261395

[87] Angum F., Khan T., Kaler J., Siddiqui L., Hussain A., The Prevalence of Autoimmune Disorders in Women: A Narrative Review. Cureus 12, e8094 (2020). 32542149 10.7759/cureus.8094 PMC7292717

[88] Desai M. K., Brinton R. D., Autoimmune Disease in Women: Endocrine Transition and Risk Across the Lifespan. Front Endocrinol (Lausanne) 10, 265 (2019). 31110493 10.3389/fendo.2019.00265 PMC6501433

[89] Sziraki A., Lu Z., Computational Pipeline for Processing EasySci Data (2023; https://zenodo.org/record/8395492).

[90] Martin B. K., Qiu C., Nichols E., Phung M., Green-Gladden R., Srivatsan S., Blecher-Gonen R., Beliveau B. J., Trapnell C., Cao J., Shendure J., Optimized single-nucleus transcriptional profiling by combinatorial indexing. Nat Protoc 18, 188–207 (2023). 36261634 10.1038/s41596-022-00752-0 PMC9839601

[91] Cao J., O’Day D. R., Pliner H. A., Kingsley P. D., Deng M., Daza R. M., Zager M. A., Aldinger K. A., Blecher-Gonen R., Zhang F., Spielmann M., Palis J., Doherty D., Steemers F. J., Glass I. A., Trapnell C., Shendure J., A human cell atlas of fetal gene expression. Science 370 (2020). 10.1126/science.aba7721 PMC7780123 33184181

[92] McInnes L., Healy J., Melville J., UMAP: Uniform Manifold Approximation and Projection for Dimension Reduction, arXiv [stat.ML] (2018). http://arxiv.org/abs/1802.03426.

[93] Hao Y., Hao S., Andersen-Nissen E., Mauck W. M. 3rd, Zheng S., Butler A., Lee M. J., Wilk A. J., Darby C., Zager M., Hoffman P., Stoeckius M., Papalexi E., Mimitou E. P., Jain J., Srivastava A., Stuart T., Fleming L. M., Yeung B., Rogers A. J., McElrath J. M., Blish C. A., Gottardo R., Smibert P., Satija R., Integrated analysis of multimodal single-cell data. Cell 184, 3573–3587.e29 (2021). 34062119 10.1016/j.cell.2021.04.048 PMC8238499

[94] Zhang Y., Liu T., Meyer C. A., Eeckhoute J., Johnson D. S., Bernstein B. E., Nusbaum C., Myers R. M., Brown M., Li W., Liu X. S., Model-based analysis of ChIP-Seq (MACS). Genome Biol. 9, R137 (2008). 18798982 10.1186/gb-2008-9-9-r137 PMC2592715

[95] Schug J., Schuller W.-P., Kappen C., Salbaum J. M., Bucan M., Stoeckert C. J. Jr, Promoter features related to tissue specificity as measured by Shannon entropy. Genome Biol 6, R33 (2005). 15833120 10.1186/gb-2005-6-4-r33 PMC1088961

[96] Finucane H. K., Reshef Y. A., Anttila V., Slowikowski K., Gusev A., Byrnes A., Gazal S., Loh P.-R., Lareau C., Shoresh N., Genovese G., Saunders A., Macosko E., Pollack S., Brainstorm Consortium, Perry J. R. B., Buenrostro J. D., Bernstein B. E., Raychaudhuri S., McCarroll S., Neale B. M., Price A. L., Heritability enrichment of specifically expressed genes identifies disease-relevant tissues and cell types. Nat Genet 50, 621–629 (2018). 29632380 10.1038/s41588-018-0081-4 PMC5896795

[97] Cusanovich D. A., Hill A. J., Aghamirzaie D., Daza R. M., Pliner H. A., Berletch J. B., Filippova G. N., Huang X., Christiansen L., DeWitt W. S., Lee C., Regalado S. G., Read D. F., Steemers F. J., Disteche C. M., Trapnell C., Shendure J., A Single-Cell Atlas of In Vivo Mammalian Chromatin Accessibility. Cell 174, 1309–1324.e18 (2018). 30078704 10.1016/j.cell.2018.06.052 PMC6158300

[98] Sollis E., Mosaku A., Abid A., Buniello A., Cerezo M., Gil L., Groza T., Güneş O., Hall P., Hayhurst J., Ibrahim A., Ji Y., John S., Lewis E., MacArthur J. A. L., McMahon A., Osumi-Sutherland D., Panoutsopoulou K., Pendlington Z., Ramachandran S., Stefancsik R., Stewart J., Whetzel P., Wilson R., Hindorff L., Cunningham F., Lambert S. A., Inouye M., Parkinson H., Harris L. W., The NHGRI-EBI GWAS Catalog: knowledgebase and deposition resource. Nucleic Acids Res 51, D977–D985 (2023). 36350656 10.1093/nar/gkac1010 PMC9825413

[99] Stuart T., Srivastava A., Madad S., Lareau C. A., Satija R., Single-cell chromatin state analysis with Signac. Nat Methods 18, 1333–1341 (2021). 34725479 10.1038/s41592-021-01282-5 PMC9255697

[100] Castro-Mondragon J. A., Riudavets-Puig R., Rauluseviciute I., Lemma R. B., Turchi L., Blanc-Mathieu R., Lucas J., Boddie P., Khan A., Manosalva Pérez N., Fornes O., Leung T. Y., Aguirre A., Hammal F., Schmelter D., Baranasic D., Ballester B., Sandelin A., Lenhard B., Vandepoele K., Wasserman W. W., Parcy F., Mathelier A., JASPAR 2022: the 9th release of the open-access database of transcription factor binding profiles. Nucleic Acids Res 50, D165–D173 (2022). 34850907 10.1093/nar/gkab1113 PMC8728201

[101] Gu Z., Eils R., Schlesner M., Complex heatmaps reveal patterns and correlations in multidimensional genomic data. Bioinformatics 32, 2847–2849 (2016). 27207943 10.1093/bioinformatics/btw313

[7] Zhang Z., Schaefer C., Jiang W., Lu Z., Lee J., Sziraki A., Abdulraouf A., Wick B., Haeussler M., Li Z., Molla G., Satija R., Zhou W., Cao J., A panoramic view of cell population dynamics in mammalian aging. Science 387, eadn3949 (2025). 39607904 10.1126/science.adn3949 PMC11910726

