# Supplementary material for: Organism-wide cellular dynamics and epigenomic remodeling in mammalian aging
Source: bioRxiv. 2025 May 15:2025.05.12.653376. Preprint. [Version 1] doi: 10.1101/2025.05.12.653376 (PMC12132170; doi:10.1101/2025.05.12.653376)
Supplement: Supplement 2 [file NIHPP2025.05.12.653376v1-supplement-2.pdf]

bioRxiv preprint doi: https://doi.org/10.1101/2025.05.12.653376; this version posted May 15, 2025. The copyright holder for this preprint (which was not certified by peer review) is the author/funder, who has granted bioRxiv a license to display the preprint in perpetuity. It is made available under aCC-BY 4.0 International license.

Supplementary Materials

Materials and Methods:

Animals and organ collection

The C57BL/6 wild-type mice at one month (n=10, 5 males and 5 females), five months (n=10, 5 males and 5 females), and twenty-one months (n=12, 6 males and 6 females) were obtained from The Jackson Laboratory. Detailed information on animal individuals in this study is provided in Table S1A. Mice were housed socially and maintained on a regular 12h/12h day/night cycle. In order to analyze newborn cells from the same set of samples in parallel, all mice were labeled with EdU (25 mg/kg, i.p. injection) at 24-hour intervals for five days. Tissues were harvested one day after the final injection. To control for circadian effects, sample harvest was performed around the same period (4-7 PM) across all individuals. Mice were euthanized utilizing inhalation of carbon dioxide (CO2). After euthanization, major organs were quickly transferred into ice-cold PBS, and the following tissues were collected: brown adipose tissue, bone marrow, cecum, colon, esophagus, eye, heart, small intestine (spanning duodenum, jejunum, and ileum), kidney, liver, lung, muscle (quadriceps and gastrocnemius), ovary and uterus, pancreas, back skin, spleen, stomach, thymus, gonadal white adipose tissue, inguinal white adipose tissue, mesenteric adipose tissue. Dissected mouse tissues were snap-frozen in liquid nitrogen and stored at -80C. All animal procedures were in accordance with institutional regulations under the IACUC protocol 21049.

Nuclei extraction from multiple mammalian organs

Nuclei extraction was performed using the method from (*90*) and (*7*) with slight modifications. Before extraction, frozen tissues were placed inside aluminum foil, and smashed into powders on dry ice with a hammer. 10X PBS-hypotonic stock solution was prepared as follows: 6.83 g of Na_2HPO_4-$2H_2O$ (Sigma, 71643-250G), 3.5 g of NaH_2PO_4-H_2O (CATALOG), 1.2 g of KH_2PO_4 (Sigma, P285-500), 1 g of KCl (Sigma, P9541-1KG) and 3 g of NaCl (Sigma, P9888-500G) in nuclease-free water to a final volume of 500 mL. On the day of nuclei extraction, 1X nuclei lysis buffer was prepared freshly as follows: final concentration of 1X PBS-hypotonic stock solution, 3 mM $MgCl_2$ (VWR, 97062-848), 0.025% IGEPAL CA-630 (VWR, IC0219859650), 0.1% Tween-20 (Sigma, P9416-100ML), 1X cOmplete, EDTA-free Protease Inhibitor Cocktail (Sigma, 11873580001). In addition, a final concentration of 0.33M sucrose (Sigma, S0389) was included in the lysis buffer for these tissues: esophagus, stomach, intestinal, cecum, colon, spleen, thymus, bone marrow and skin. The powdered tissues were then transferred into 10-20 mL nuclei lysis buffer, followed by brief vortexing and incubation for 10-15 minutes at 4C on a rotator. Extracted nuclei were then filtered through 40 um cell strainers (VWR, 470236-276), stained with 4',6-diamidino-2-phenylindole staining (DAPI, Invitrogen D1306), and FACS sorted for singlets.

EasySci library construction and sequencing

The EasySci-ATAC library was prepared following the prior study (*15*), except for a few modifications. The sorted nuclei were pelleted down at 500g for 5 minutes, resuspend in nuclei buffer [10 mM Tris-HCl pH 7.5 (VWR, 97062-936), 10 mM NaCl (VWR, 97062-858), 3 mM $MgCl_2$ (VWR, 97062-848), 0.025% IGEPAL CA-630 (VWR, IC0219859650), 0.1% Tween-20 (Sigma, P9416-100ML), 1X cOmplete, EDTA-free Protease Inhibitor Cocktail (Sigma, 11873580001)] to a final concentrations of 1000~2000 nuclei/μL. Nuclei were mixed in a 1:1 ratio with 2X TD buffer [20 mM Tris-HCl pH 7.5, 20 mM $MgCl_2$, 20% Dimethylformamide (Fisher, AC327175000)] and dispensed 10 μL into each well of four 96-well plates. 1 μL barcoded Tn5 was

bioRxiv preprint doi: https://doi.org/10.1101/2025.05.12.653376; this version posted May 15, 2025. The copyright holder for this preprint (which was not certified by peer review) is the author/funder, who has granted bioRxiv a license to display the preprint in perpetuity. It is made available under aCC-BY 4.0 International license.

loaded into each well. Tagmentation reaction was performed at 37°C for 30 minutes with gentle shaking at 300 rpm and stopped by adding 11 μL of 2X Stop buffer [40 mM EDTA (VWR, 37062-656), 1 mM Spermidine (Sigma, S0266-1G)] to each well. Samples were pooled and washed twice and resuspended resuspended in nuclei buffer. 5 μL of resuspended nuclei were distributed into each well of 96-well plates. 2 μL indexed EasySci P5 ligation adapters and 3 μL ligation mix [1 μL nuclease-free water, 1 μL 10X T4 DNA ligase buffer, 1 μL T4 DNA ligase (NEB, M0202L)] were added to each well. Ligation was performed at room temperature for 30 minutes with medium-speed rocking (350g) and stopped by adding 2 μL of 18 mM EDTA to each well. After that, nuclei were pooled, washed, resuspended using nuclei buffer, and subjected to another round of FAC sort based on DAPI staining to remove doublets. Then, sorted nuclei were distributed into PCR plates as 5 uL per well. Proteinase K treatment was performed by mixing each well with 0.25 μL 18.9 mg/mL proteinase K (Sigma, 3115828001), 0.25 μL 1% SDS, and 0.5 μL EB buffer, and plates were incubated at 65°C for 16 hours. Then, 2 μL 10% Tween-20 was added to each well to quench the SDS. Following on, 1 μL of 10 μM universal P5 primer (5′-AATGATACGGCGACCACCGAGATCTACAC-3′, IDT), 1 μL of 10 μM indexed P7 primer (5'-CAAGCAGAAGACGGCATACGAGAT[i7]GTGACTGGAGTTCAGACGTGTGCTCTTCCGATCT-3', IDT) and 10 μL NEBNext High-Fidelity 2X PCR Master Mix (NEB M0541L) were added into each well. Amplification was carried out using the following program: 72°C for 5 minutes, 98°C for 30 seconds, 13-14 cycles of 98°C for 10 seconds, 66°C for 30 seconds, 72°C for 30 seconds and a final 72°C for 5 minutes. Final PCR products were pooled and purified by column purification using a Zymo DNA Clean & Concentrator kit (Zymoresearch, D4014) followed by gel extraction using Zymoclean Gel DNA Recovery Kit (Zymoresearch, D4007) to remove adapter dimers. Library concentrations were determined by Qubit and the libraries were visualized by electrophoresis on a 2% E-Gel™ EX Agarose Gels (Invitrogen G402022).

Processing of sequencing reads

Base calls were converted to fastq format and demultiplexed using Illumina's bcl2fastq/v2.19.0.316 tolerating one mismatched base in barcodes (edit distance (ED) <= 1). Then, indexed Tn5 barcodes and ligation barcodes were extracted, and corrected to their nearest barcode (edit distance (ED) <= 1). Reads with uncorrected barcodes (ED >= 2) were removed. Tn5 adaptors were removed from 5'-end and clipped from 3'-end using trim_galore/0.4.1 (https://github.com/FelixKrueger/TrimGalore). Trimmed reads were mapped to the mouse genome (mm10) using STAR/v2.5.2b with default settings. Aligned reads were filtered using samtools/v1.4.1 to retain reads mapped in proper pairs with quality score MAPQ > 30 and to keep only the primary alignment. Duplicates were removed by picard MarkDuplicates/v2.25.2 per PCR sample. Deduplicated bam files were converted to bedpe format using bedtools/v2.30.0, which were further converted to offset-adjusted (+4 bp for plus strand and -5 bp for minus) fragment files (.bed). Deduplicated reads were further split into constituent cellular indices using the Tn5 and ligation barcodes, and sparse matrices counting reads overlapping with promoter regions (±1 kb around transcription start site) were generated for quality filtering. In the meantime, fragment files were used to generate h5ad files for all downstream analyses with the snap.pp.import_data() function of SnapATAC2/v2.5.1. Cell-by-bin matrices were also generated with SnapATAC2 function snap.pp.add_tile_matrix() containing insertion counts across genome-wide 5000-bp bins.

Cell filtering, dimensionality reduction, clustering, and annotations

The following analyses were performed on the dataset of each tissue separately. After initial processing, cells with less than 1000 unique reads or less than 15% of reads in promoter regions were discarded. The

bioRxiv preprint doi: https://doi.org/10.1101/2025.05.12.653376; this version posted May 15, 2025. The copyright holder for this preprint (which was not certified by peer review) is the author/funder, who has granted bioRxiv a license to display the preprint in perpetuity. It is made available under aCC-BY 4.0 International license.

promoter ratio cutoff was adjusted to 0.1 for the eye dataset due to the observation of a lower promoter ratio in corneal epithelial cells. Then, we used an iterative clustering strategy to detect potential doublet cells from each organ, similar to our previous study (*15*, *91*). Briefly, doublet scores were calculated for each cell using SnapATAC2 function snap.pp.scrublet(). Meanwhile, all cells of each organ were subjected to clustering and sub-clustering analysis with spectral embedding and graph-based clustering implemented in SnapATAC2. Cells labeled as doublets (defined by a doublet probability cutoff of 0.5) or from doublet-derived sub-clusters (defined by a doublet ratio cutoff of 0.2) were manually examined and filtered out. We then generated gene activity matrices for each organ by counting the Tn5 insertions in the TSS and gene body regions for each gene using SnapATAC2 function snap.pp.make_gene_matrix(). Gene activity matrices were then used for cell type annotations.

To identify clusters of cells corresponding to different cell types, we subjected cells after data cleaning to dimension reduction and Leiden clustering using SnapATAC2 functions snap.tl.spectral(), snap.pp.knn() and snap.tl.leiden() with the default setting. UMAP coordinates were calculated based on the spectral embedding matrices using the function UMAP() with min_dist=0.01 from the Python package umap/v0.5.2 (*92*). For cell annotations, we first obtained a draft of annotations by integrating our chromatin data (subsampled to 2,000 cells per Leiden cluster) with the published sn-RNA-seq datasets (*7*) through Seurat/4.3.0.1 (*7*, *93*) label transfer, and we manually reviewed and refined the annotations for each Leiden cluster based on accessibilities of known markers listed in Table S2.

Peak calling

After cell annotation, reads from each main cell type of each tissue were concatenated. Then, Tn5-corrected single-base insertions were extracted and subjected to peak calling using macs3/v3.0.0b3 (*94*), with the following parameters: --nomodel --extsize 200 --shift -100 -q 0.1. Peak summits were extended by 250bp on either side and then merged iteratively, similar to (*12*). Specifically, the peak with the smallest p-value was kept and any peak that directly overlapped with it was removed. Then, this process was repeated to the second most significant peak and so on until all peaks were either kept or removed. Interactive merging was performed first across main cell types within each tissue, and then across all tissues to generate the universal peak set across the entire organism. The peak count matrices were generated using the SnapATAC2 function snap.pp.make_peak_matrix(). Peak annotations were performed using the HOMER function annotatePeaks.pl.

Identifications of cell-type-specific peaks

We used a Shannon entropy-based method to identify cell type-specific peaks, similar to previous studies (*12*, *95*). Starting from 144 unique cell types collapsed from all tissues, we first aggregated the peak count matrix for each cell type and normalized the aggregated matrix to counts per million (CPM). We then converted the CPM values into probabilities by dividing each cell type's CPM by the total CPM across all cell types for a given peak: $p_i = q_i / \Sigma q_i$, where "q_i" is the CPM value of the i-th cell type of a given peak. Next, we calculated Shannon entropy for each peak, which measures how uniformly accessibility is distributed across cell types: $H = - \Sigma (p_i * \log(p_i))$ where "p_i" is the probability of the i-th cell type for a given peak. To determine statistical significance, we generated a background distribution of entropy values using a set of peaks with low variability. Specifically, for each peak, we calculated the fold change between the most accessible cell type (quantified by CPM) and the average accessibility across all cell types. We then grouped peaks based on their mean accessibility into thirty bins. Within each bin, peaks with fold-change values below the 25th percentile were considered low-variable peaks, while those in the top 25th

bioRxiv preprint doi: https://doi.org/10.1101/2025.05.12.653376; this version posted May 15, 2025. The copyright holder for this preprint (which was not certified by peer review) is the author/funder, who has granted bioRxiv a license to display the preprint in perpetuity. It is made available under aCC-BY 4.0 International license.

percentile were considered high-variable peaks. The entropy threshold was set at a p-value of 0.05, using the low-variability peaks as the background. Cell type-specific peaks were identified from the high-variable peak set if their entropy values fell below this threshold. Cell-type-specific peaks, along with their maximum and average accessibility values quantified by CPM, can be found in Table S3.

LDSC analysis

To estimate enrichments of heritability for human traits in cell-type-specific peaks, we applied LDSC (*96*), which takes summary statistics from a given GWAS as input and quantifies the enrichment of heritability in an annotated set of SNPs conditioned on a baseline model that accounts for the non-random distribution of heritability across the genome. The LDSC computational pipeline was modified from (*97*) and the LDSC tutorial (https://github.com/bulik/ldsc/wiki/Cell-type-specific-analyses). Specifically, we first used the UCSC utility liftOver to lift all GWAS SNPs from the human to the mouse genome. We then took the top 2000 differentially accessible peaks per main cell type identified from the entropy-based method, and annotated each SNP according to whether or not it overlapped these cell-type-specific peaks. We then followed the recommended workflow for running LDSC using HapMap SNPs, precomputed files corresponding to 1000 genomes phase 3, excluding the MHC region to generate an LDSC model for each chromosome and peak set. GWAS summary statistics were obtained from the Broad LD Hub (https://data.broadinstitute.org/alkesgroup/sumstats_formatted/) and from GWAS catalogs (*98*) (https://www.ebi.ac.uk/gwas/downloads/summary-statistics). Coefficient P values calculated from LDSC were corrected for multiple hypotheses for each trait using the Benjamini-Hochberg method.

Identifications of sex dimorphism for main cell types

To examine cell types with sex dimorphism in chromatin accessibility profiles, we trained k-nearest neighbor (KNN) classifiers to distinguish male and female cells of the same age for each main cell type using python package sklearn/v1.0.2. Spectral embeddings generated from SnapATAC2 were used as input features to predict the sex of individual cells. Datasets were filtered to include only cell types with at least 200 total cells and a minimum of 50 cells per sex. We performed five independent train-test splits, using 80% of the data for training and 20% for testing, with stratified sampling to maintain sex balance. To ensure computational efficiency, we subsampled both training and test sets to a maximum of 2000 cells per cell type. Classification performance was evaluated using area under the curve (AUC) metrics, with cell types exhibiting AUC > 0.9 considered to display strong sex-associated chromatin differences.

Sub-clustering analysis

Sub-clustering analysis was performed using SnapATAC2, same as main clustering. Briefly, we subjected cells of the same type within the same tissue to feature selection (n_features=50000), dimension reduction and leiden clustering (resolution=1.5). UMAP coordinates were calculated based on the spectral embedding matrices (min_dist=0.01). We then examined the resulting Leiden clusters and manually merged those that overlapped in UMAP space without clear differences in gene accessibility. For certain cell types with pronounced sex dimorphism, including liver hepatocytes, kidney proximal tubule cells, type IIB myonuclei in muscle, adipocytes, and adipose stem and progenitor cells from gonadal white adipose tissue, sub-clustering was performed separately for males and females to capture cell-state differences rather than sex-based differences.

Differential Abundance Analysis

bioRxiv preprint doi: https://doi.org/10.1101/2025.05.12.653376; this version posted May 15, 2025. The copyright holder for this preprint (which was not certified by peer review) is the author/funder, who has granted bioRxiv a license to display the preprint in perpetuity. It is made available under aCC-BY 4.0 International license.

For each mouse individual, we quantified the proportion of each main cell type or subtype within each tissue, and applied a linear regression model (proportion ~ age + age:sex) to assess age-associated population dynamics while accounting for sex effects. The analysis was conducted using the R function lm(). Quadriceps and gastrocnemius muscles were analyzed separately, as they were from distinct anatomical locations. In the model, age was treated as a continuous variable (in months), while sex was considered a binary categorical variable. To identify significantly changing subtypes, we filtered results based on the following criteria: $R^2 > 0.4$ and a q-value (for either the age term or the age:sex interaction term) < 0.05. Multiple hypothesis correction was performed separately for each tissue using the Benjamini-Hochberg method. Additionally, Pearson correlation coefficients were calculated between the proportion of each main cell type or subtype and age. Based on these correlations, cell types and subtypes were classified into three groups: aging-up (q-value of the age term < 0.05, q-value of the age:sex interaction > 0.05, Pearson r > 0), aging-down (q-value of the age term < 0.05, q-value of the age:sex interaction > 0.05, Pearson r < 0), interaction-significant (q-value of the age:sex interaction < 0.05).

Differential Peak Analysis

In order to reduce the search space for differential peak analysis, we first selected highly accessible and highly variable peaks for each main cell type. First, the accessibility levels of all 1.3M peaks for each condition were quantified by counts per million (CPM) after aggregating data of the same age. Peaks were then ranked based on their CPM value in the most accessible condition, and only those above the 75th percentile were retained for each main cell type. These highly accessible peaks were further grouped into thirty bins based on their maximum accessibility. Within each bin, peaks with fold-change values (max condition vs. mean of three age groups) in the top 25th percentile were classified as highly variable peaks. This preselection was conducted separately for males and females, and the resulting peaks were collapsed across sexes.

Pre-filtered peaks for each main cell type were then analyzed for differential accessibility using edgeR/v3.36.0 (*12*, *77*). The analysis was performed at the pseudo-bulk level by aggregating single-cell peak counts from the same mouse sample, treating each individual mouse as a replicate. Age was treated as a continuous variable (in months). Only cell types with more than 200 cells in all three age groups were included in the analysis. Additionally, male and female samples were tested separately, with log fold-change (logFC), p-values, and q-values computed for each sex independently using edgeR. Differentially accessible peaks were categorized as follows: sex-shared, aging up-regulated peaks (p-value-female < 0.05 and p-value-male < 0.05, q-value-male < 0.05 or q-value-male < 0.05, logFC-female > 0, logFC-male > 0); sex-shared, aging down-regulated peaks (p-value-female < 0.05 and p-value-male < 0.05, q-value-male < 0.05 or q-value-male < 0.05, logFC-female < 0, logFC-male < 0); female-specific DA peaks (p-value-male > 0.05, q-value-female < 0.05); male-specific DA peaks (p-value-female > 0.05, q-value-male < 0.05).

Identifying aging-associated linkages of genes, promoters and cis-regulatory elements

This analysis aims to identify links between cis-regulatory elements and their putative target genes, and to validate accessibility changes with gene expression data. First, we focused on aging-associated differentially accessible (DA) peaks identified previously. Pearson correlation coefficients were computed between DA promoters and nearby non-promoter DA peaks (±500 kb) across samples, after pseudo-bulking each mouse individual. A background distribution was also generated by pairing DA promoters

bioRxiv preprint doi: https://doi.org/10.1101/2025.05.12.653376; this version posted May 15, 2025. The copyright holder for this preprint (which was not certified by peer review) is the author/funder, who has granted bioRxiv a license to display the preprint in perpetuity. It is made available under aCC-BY 4.0 International license.

with randomly selected peaks, and a threshold of Pearson correlation was defined with an empirical FDR = 0.05. The correlation analyses were performed separately for sex-shared, female-specific and male-specific peaks. In the meantime, we collected snRNA-seq data of mouse aging from (*7*), re-annotated the cells for consistent cell type labels between RNA-seq and ATAC-seq, and followed with differential gene expression changes using edgeR/v3.36.0 (*77*). Similar to differential accessible analysis, the differential expression analysis was performed separately for males and females in terms of each main cell type in each tissue, and at the pseudo-bulk level by aggregating single-cell gene counts from the same mouse individual. Differentially expressed genes, including sex-shared (p-value < 0.1 in both males and females, with the same logFC direction), female-specific (p-value < 0.1 in females but > 0.1 in males) and male-specific changes (p-value < 0.1 in males but > 0.1 in females) were defined. Finally, aging-associated linkages (gene-promoter-CRE) were defined if a gene exhibited consistent changes (up-regulated or down-regulated) in the same cell type from the same tissue across three layers, i.e, expression changes based on RNA-seq data, accessibility changes of promoters and non-promoter peaks based on ATAC-seq data, significant correlations between accessibilities of promoters and linked non-promoter sites.

Transcription factor motif analysis

We used Signac/v1.7.0 (*99*) and chromVAR/v1.16.0 (*21*) to quantify motif accessibility of transcription factors in Figure 3N, 5C, 5I, S3E, S3F and S7B. Position weight matrices of transcription factor binding sites were obtained from JASPAR2022 (*100*). A Signac object containing a peak count matrix of cells of interest was constructed using the Signac functions CreateChromatinAssay() and CreateSeuratObject(). Motif information was then added via AddMotifs(), and motif deviation scores were calculated for each single cell using RunChromVAR(). In Figure S3F, to compare motif activity and gene accessibility of transcription factors across cell types, motif deviation scores were rescaled to the range (0,10) using the R function rescale(), averaged per cell type, and scaled to z-score across cell types. In Figure 5C, motif footprinting analysis was performed using the Footprint() function in Signac.

For motif enrichment analysis of aging-associated differentially accessible (DA) peaks (Figure 7A, 7C, 7D, and 7E), we used the HOMER function findMotifsGenome.pl (*73*). A background peak set with minimal age-related changes was selected for each main cell type as a control for HOMER analysis. Specifically, accessible peaks for each cell type were grouped into thirty bins, based on accessibility in the highest condition. Within each bin, peaks with fold-change values (maximum condition vs. the mean of three age groups) in the bottom 10th percentile were classified as invariable peaks. The same background peak set was used to assess transcription factor motif enrichment across all aging-associated peak sets of a given cell type. Motif enrichments were quantified as the percentage of peaks containing a given motif in the target peak set relative to the background. Significance values (-log10 q-value) were calculated within HOMER.

Cytokine-related analysis

To evaluate the contribution of cytokine signaling to age-related molecular changes in B cells and macrophages, aging-upregulated DA peaks of each sex identified from chromatin accessibility data were first collapsed to their nearest genes using the HOMER function annotatePeaks.pl. The resulting gene sets (female-upregulated and male-upregulated) were compared to cytokine-induced gene sets of the same cell type from the Immune Dictionary Data (*82*). A hypergeometric test was performed using the R function phyper(). We only kept conditions containing more than ten DE genes after cytokine treatments identified from Immune Dictionary for this comparison.

bioRxiv preprint doi: https://doi.org/10.1101/2025.05.12.653376; this version posted May 15, 2025. The copyright holder for this preprint (which was not certified by peer review) is the author/funder, who has granted bioRxiv a license to display the preprint in perpetuity. It is made available under aCC-BY 4.0 International license.

As an additional measure of aging signatures across cytokine treatments, we overlapped the aging-upregulated gene signatures from our study with the union of genes that exhibited increased expression in at least one cytokine treatment. This overlapping gene set represents shared features between aging and cytokine responses. To identify cytokines that mimic aging phenotypes, we compared the aggregated expression of these genes across cytokine treatments to the PBS control. Statistical evaluation was conducted using Wilcoxon rank-sum tests (wilcox.test() in R).

To minimize cross-activation effects, we excluded cytokines whose receptors were not expressed (TPM < 1) in the target cell type, based on gene expression profiles from the Immune Dictionary. To further validate cytokine signaling changes and refine potential targets, we further analyzed chromatin accessibility changes in genes encoding cytokines and their receptors. Differential analyses were performed using gene accessibility matrices at the pseudo-bulk level, with females and males analyzed separately.

Finally, cytokine signatures were defined based on the following criteria: significant overlap with aging-peak-associated genes (p-value < 0.05, hypergeometric test); significant aging-mimicking activation compared to PBS (p-value < 0.05, Wilcoxon rank-sum test); can be supported by increased chromatin accessibility of the underlying genes in at least one cell type within the same tissue, or from increased chromatin accessibility of receptor genes in the target cell type.

bioRxiv preprint doi: https://doi.org/10.1101/2025.05.12.653376; this version posted May 15, 2025. The copyright holder for this preprint (which was not certified by peer review) is the author/funder, who has granted bioRxiv a license to display the preprint in perpetuity. It is made available under aCC-BY 4.0 International license.

Supplementary figures

Figs. S1 to S10

bioRxiv preprint doi: https://doi.org/10.1101/2025.05.12.653376; this version posted May 15, 2025. The copyright holder for this preprint (which was not certified by peer review) is the author/funder, who has granted bioRxiv a license to display the preprint in perpetuity. It is made available under aCC-BY 4.0 International license.

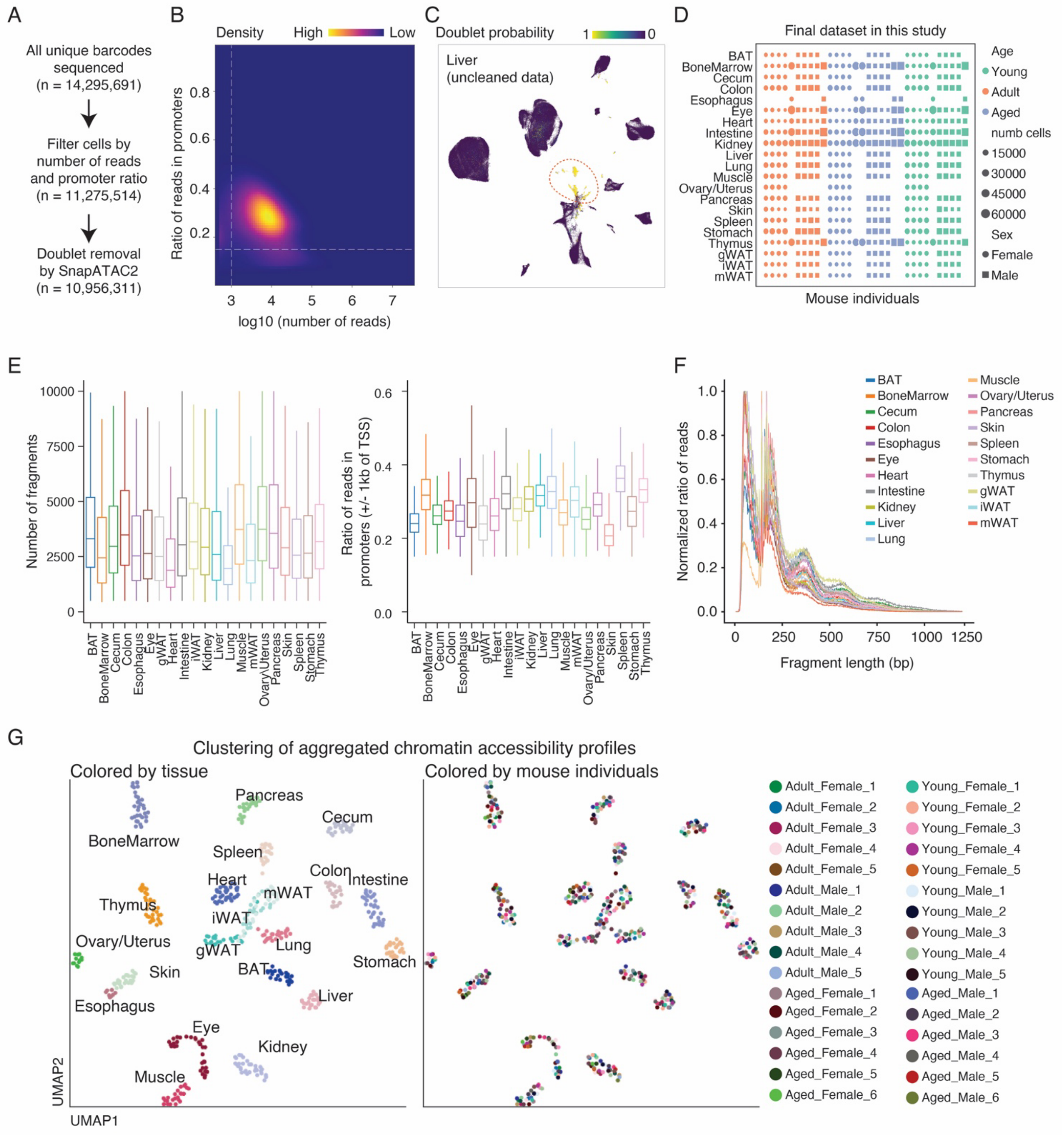

bioRxiv preprint doi: https://doi.org/10.1101/2025.05.12.653376; this version posted May 15, 2025. The copyright holder for this preprint (which was not certified by peer review) is the author/funder, who has granted bioRxiv a license to display the preprint in perpetuity. It is made available under aCC-BY 4.0 International license.

Figure S1. Overview of dataset quality.

(A) Scheme of data cleaning procedures. All sequenced cells underwent initial filtering determined by reads number and promoter ratio, followed by doublet removal using a modified pipeline based on SnapATAC2 (*17*).

(B) Density plot showing the distribution of the number of reads per nucleus versus the ratio of reads in promoters. Dash lines indicated cutoffs used for filtering (promoter ratio > 0.15 and > 1,000 unique reads).

(C) An example UMAP plot showing all liver cells (before doublet removal), colored by doublet probability. Circle indicated doublet cells.

(**D**) Dot plot showing the total number of cells obtained from each mouse individual after all quality control filterings.

(E) Box plot showing the number of fragments (left) and the ratio of reads mapped to promoters (± 1kb of TSS, right) per nucleus across tissues.

(**F**) Line plot showing the fragment length distributions of aggregated single cell ATAC-seq data across tissues.

(G) UMAP visualization of the aggregated chromatin accessibility profiles from all samples across tissues, colored by organ (left) and mouse individuals (right).

bioRxiv preprint doi: https://doi.org/10.1101/2025.05.12.653376; this version posted May 15, 2025. The copyright holder for this preprint (which was not certified by peer review) is the author/funder, who has granted bioRxiv a license to display the preprint in perpetuity. It is made available under aCC-BY 4.0 International license.

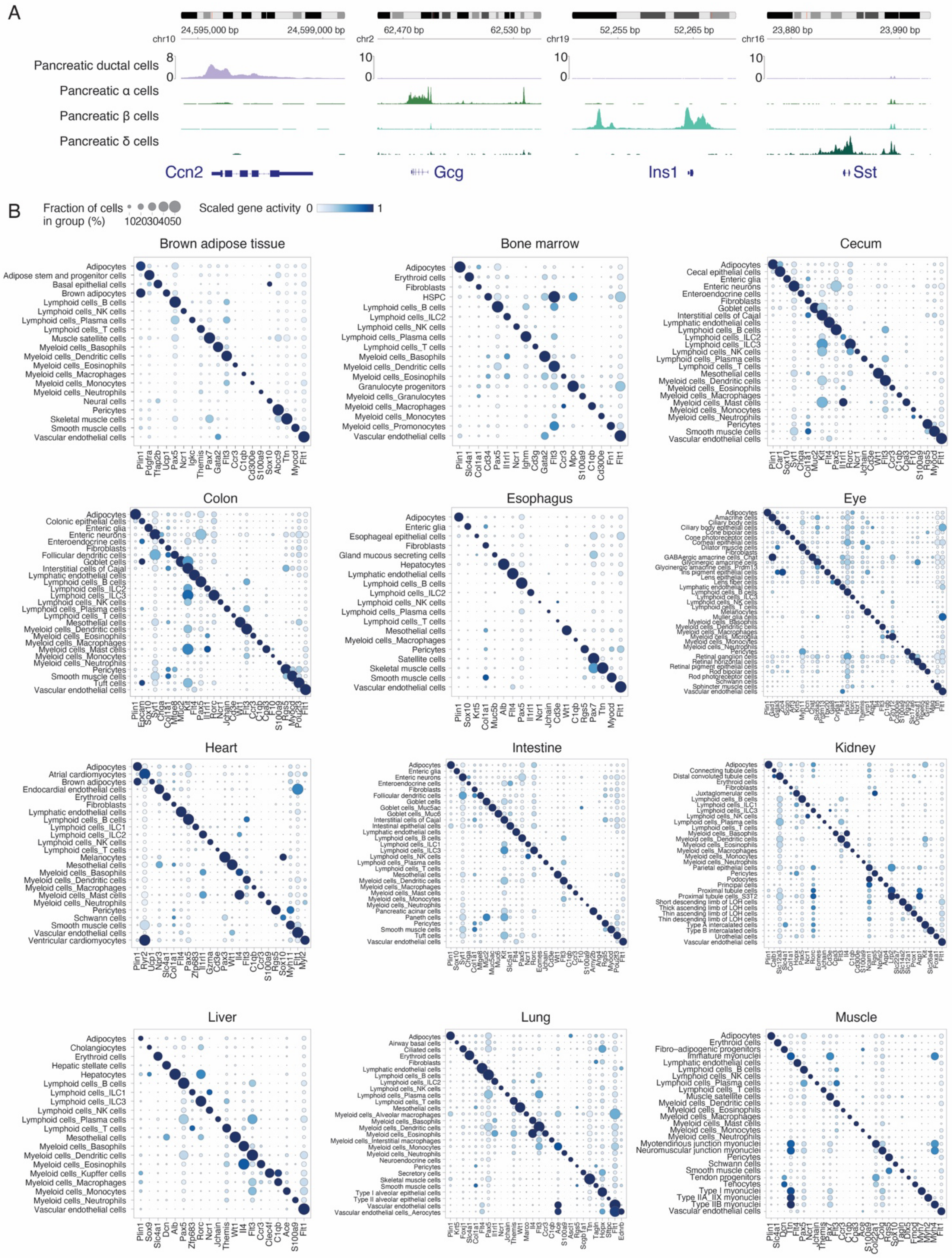

bioRxiv preprint doi: https://doi.org/10.1101/2025.05.12.653376; this version posted May 15, 2025. The copyright holder for this preprint (which was not certified by peer review) is the author/funder, who has granted bioRxiv a license to display the preprint in perpetuity. It is made available under aCC-BY 4.0 International license.

Figure S2. Annotation of main cell types using gene accessibilities.

(A) Genomic tracks showing examples of cell type annotation using accessibilities of marker genes for pancreatic ductal cells (*Ccn2*), alpha cells (*Gcg*), beta cells (*Ins1*), and delta cells (*Sst*).

(B) Dot plots showing gene markers used for annotating main cell types across tissues. The size of the dot encodes the percentage of cells within a cell type in which that marker was detected, and its color encodes the average accessibility level. A complete list of markers can be found in **Table S2**.

bioRxiv preprint doi: https://doi.org/10.1101/2025.05.12.653376; this version posted May 15, 2025. The copyright holder for this preprint (which was not certified by peer review) is the author/funder, who has granted bioRxiv a license to display the preprint in perpetuity. It is made available under aCC-BY 4.0 International license.

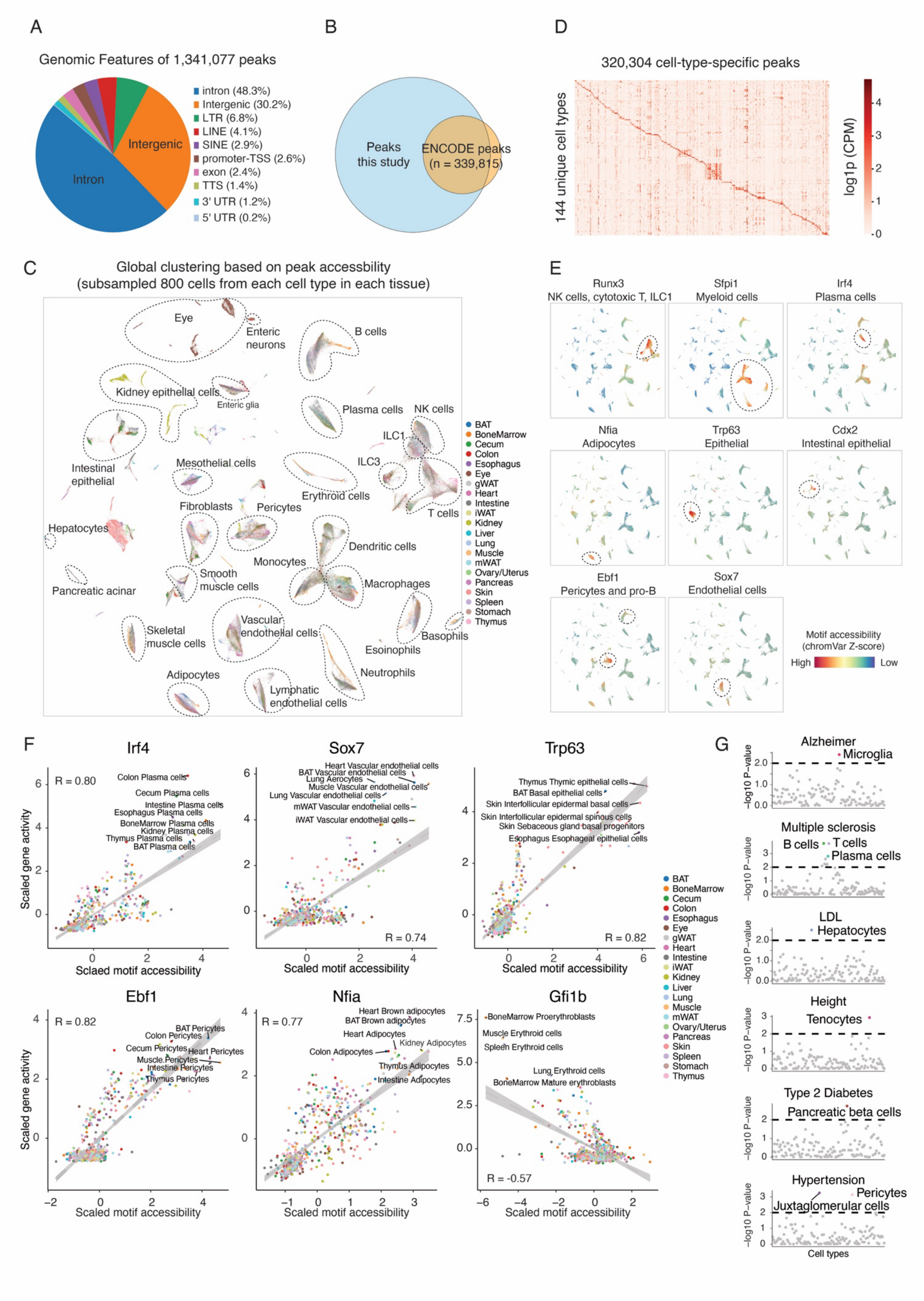

bioRxiv preprint doi: https://doi.org/10.1101/2025.05.12.653376; this version posted May 15, 2025. The copyright holder for this preprint (which was not certified by peer review) is the author/funder, who has granted bioRxiv a license to display the preprint in perpetuity. It is made available under aCC-BY 4.0 International license.

Figure S3. Identifications and characterizations of cell-type-specific cis-regulatory elements (CREs).

(A) Genomic features of 1,341,077 peaks across the mouse genome. Peaks were annotated using HOMER. TSS, transcription start site; TTS, transcription termination site; UTR, untranslated region.

(B) Venn plot showing the overlap between the peak set determined in this study and from the mouse ENCODE registry (*20*).

(C) UMAP visualization of the entire dataset subsampled to a maximum of 800 cells per main cell type per tissue, colored by the tissue type. Dimension reduction was performed using the peak-count matrix. The same cell types from multiple tissues that clustered together were circled.

(D) Heatmap showing the aggregated accessibility of peaks specific to each main cell type, quantified by counts per million.

(E) UMAP plots as in (C), colored by motif accessibilities (quantified by chromVar (*21*)) of the lineage-specific transcription factor.

(F) Scatter plots showing the example TFs whose gene accessibility levels are positively or negatively correlated with motif accessibility across cell types and tissues. Each point indicates a cell type from a specific tissue. Gene accessibilities were quantified as counts per million, and motif activities were quantified by chromVar (*21*).

(G) Scatter plots showing the enrichments of the phenotype-associated SNPs in cell-type-specific peaks. X-axis: 144 unique cell types collapsed across tissues; y-axis: significance of enrichments (-log10 p-value). Top enriched cell types were labeled for each phenotype.

bioRxiv preprint doi: https://doi.org/10.1101/2025.05.12.653376; this version posted May 15, 2025. The copyright holder for this preprint (which was not certified by peer review) is the author/funder, who has granted bioRxiv a license to display the preprint in perpetuity. It is made available under aCC-BY 4.0 International license.

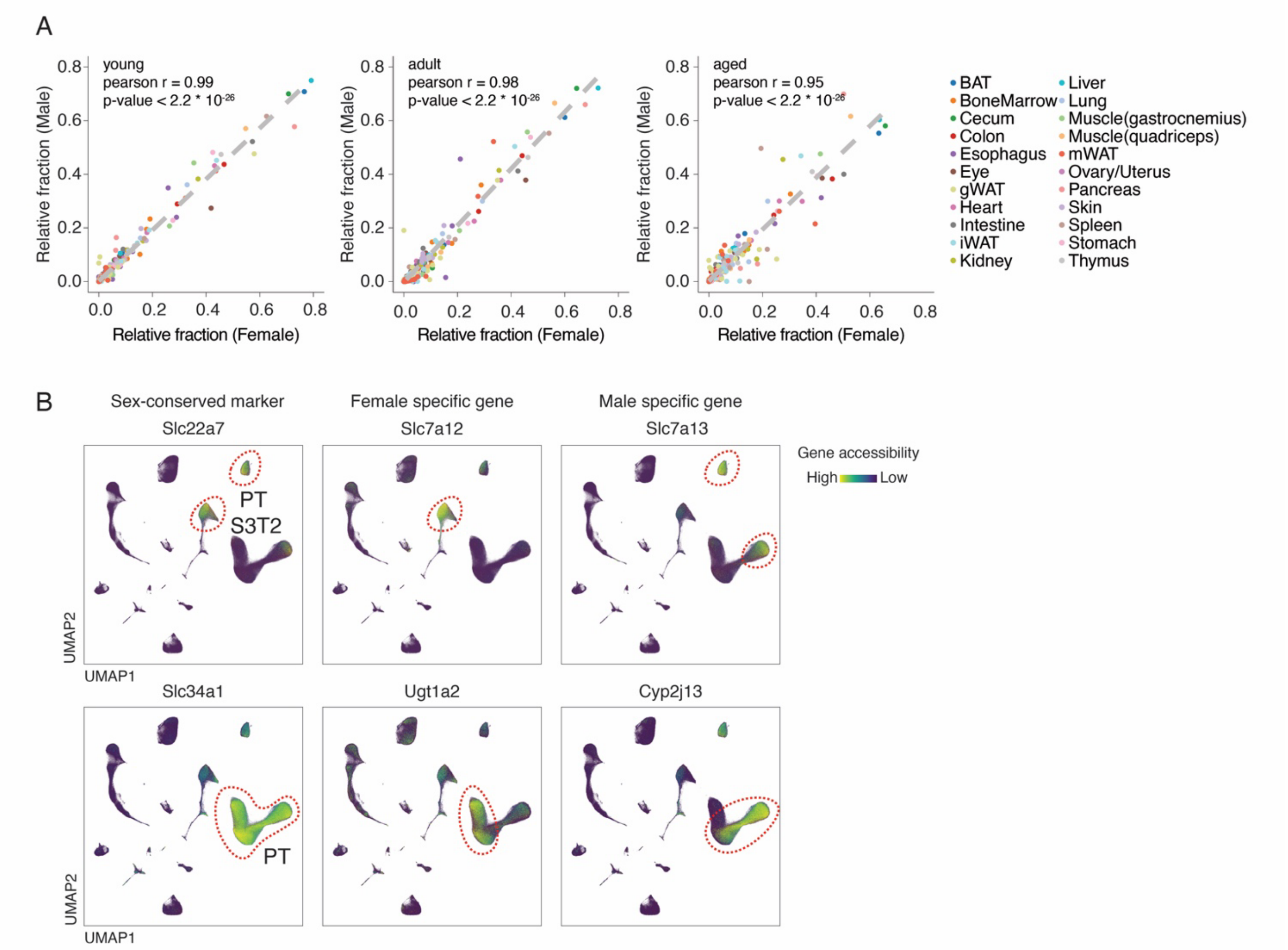

Figure S4. Examinations of sex dimorphism in cell proportion and molecular states.
(**A**) Scatterplot showing the fraction of each main cell type in each tissue between males and females, stratified by age groups. Each dot represents a cell type in a specific tissue.
(**B**) UMAP plots of all kidney cells, colored by accessibility of genes shared between sexes (left; *Slc34a1* for general proximal tubule cells (PT), *Slc22a7* for PT S3T2), unique to females (middle) and males (right).

bioRxiv preprint doi: https://doi.org/10.1101/2025.05.12.653376; this version posted May 15, 2025. The copyright holder for this preprint (which was not certified by peer review) is the author/funder, who has granted bioRxiv a license to display the preprint in perpetuity. It is made available under aCC-BY 4.0 International license.

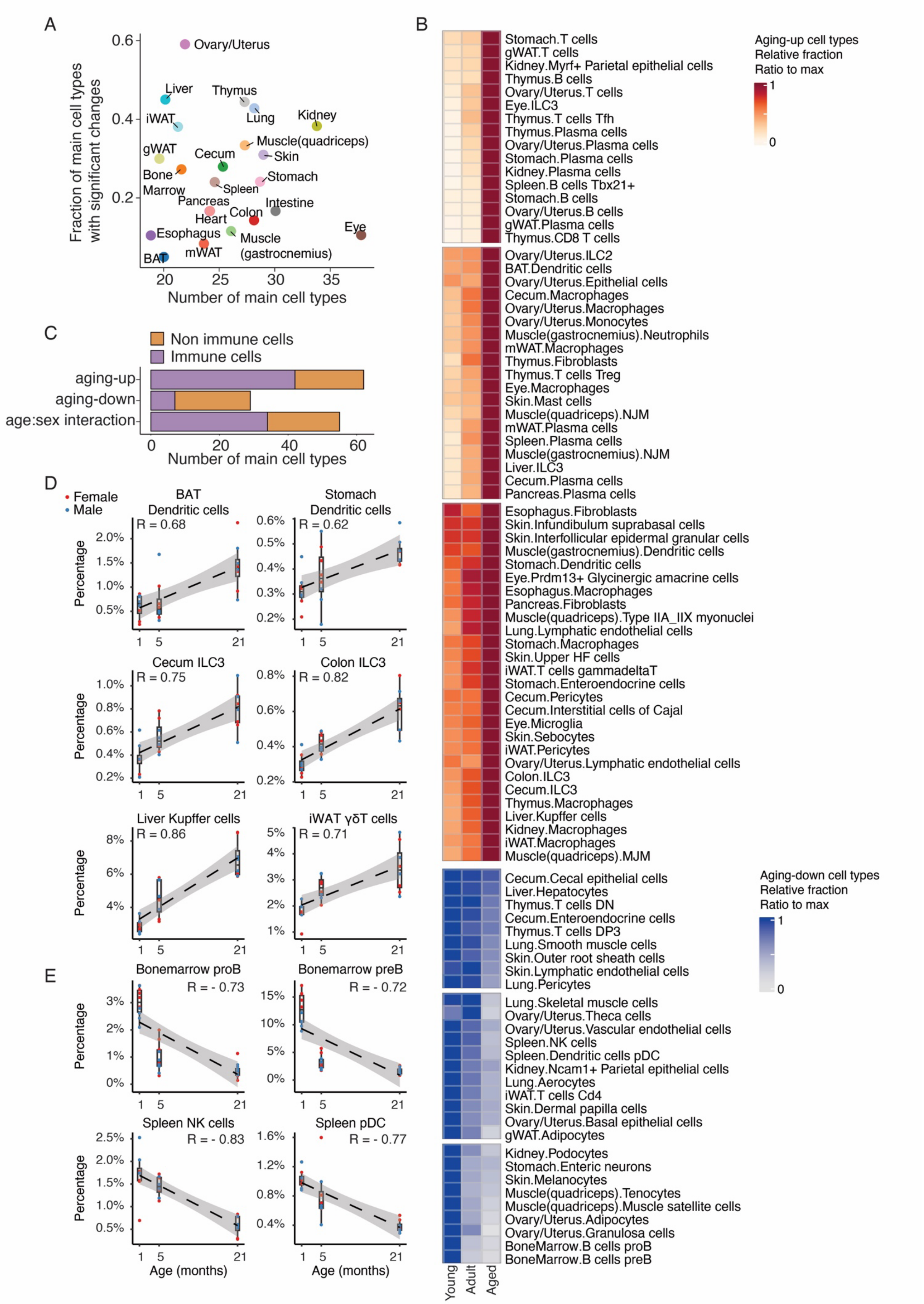

bioRxiv preprint doi: https://doi.org/10.1101/2025.05.12.653376; this version posted May 15, 2025. The copyright holder for this preprint (which was not certified by peer review) is the author/funder, who has granted bioRxiv a license to display the preprint in perpetuity. It is made available under aCC-BY 4.0 International license.

Figure S5. Aging-associated changes in main cell type proportions across tissues.

(A) Scatterplot showing the fraction of main cell types whose proportions changed significantly with age or age-sex interaction in each tissue.

(B) Heatmaps visualizing proportional changes in all aging-associated, sex-independent main cell types. The fraction of each main cell type within its tissue of origin was calculated per sample, averaged within each age group, and normalized to the most abundant condition. Cell types were ordered by hierarchical clustering implemented in ComplexHeatmap (*101*).

(C) Bar plot showing the number of main cell types derived from immune or non-immune cell types within aging-associated, sex-independent (aging-up and aging-down), or age-sex interaction groups.

(D-E) Scatter and box plots showing examples of immune cell types that expand (D) or decline (E) with age, with a linear regression line (and a Pearson correlation coefficient). Each dot represents the cell-type specific proportion of the cell type in each animal.

bioRxiv preprint doi: https://doi.org/10.1101/2025.05.12.653376; this version posted May 15, 2025. The copyright holder for this preprint (which was not certified by peer review) is the author/funder, who has granted bioRxiv a license to display the preprint in perpetuity. It is made available under aCC-BY 4.0 International license.

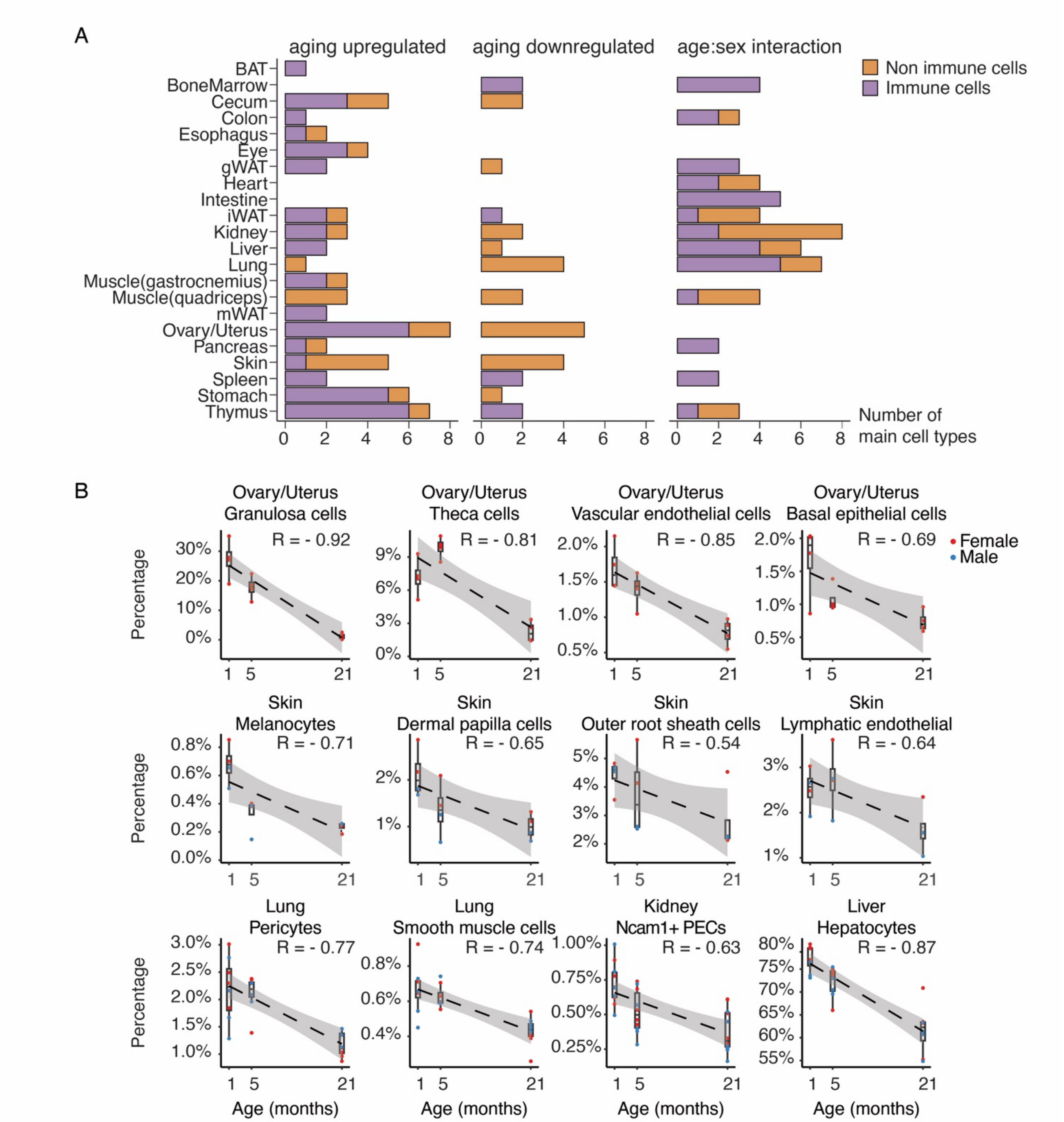

Figure S6. Aging-associated depletion of functional cell types across tissues.

(A) Bar plot showing the number of main cell types derived from immune cell types or non-immune cell types within aging-associated, sex-independent (aging-up and aging-down), or age-sex interaction groups for each tissue.

(B) Scatterplot showing examples of main cell types that decline with age across tissues, with a linear regression line (and a Pearson correlation coefficient). Each dot represents the cell-type specific proportion of the cell type in each animal.

bioRxiv preprint doi: https://doi.org/10.1101/2025.05.12.653376; this version posted May 15, 2025. The copyright holder for this preprint (which was not certified by peer review) is the author/funder, who has granted bioRxiv a license to display the preprint in perpetuity. It is made available under aCC-BY 4.0 International license.

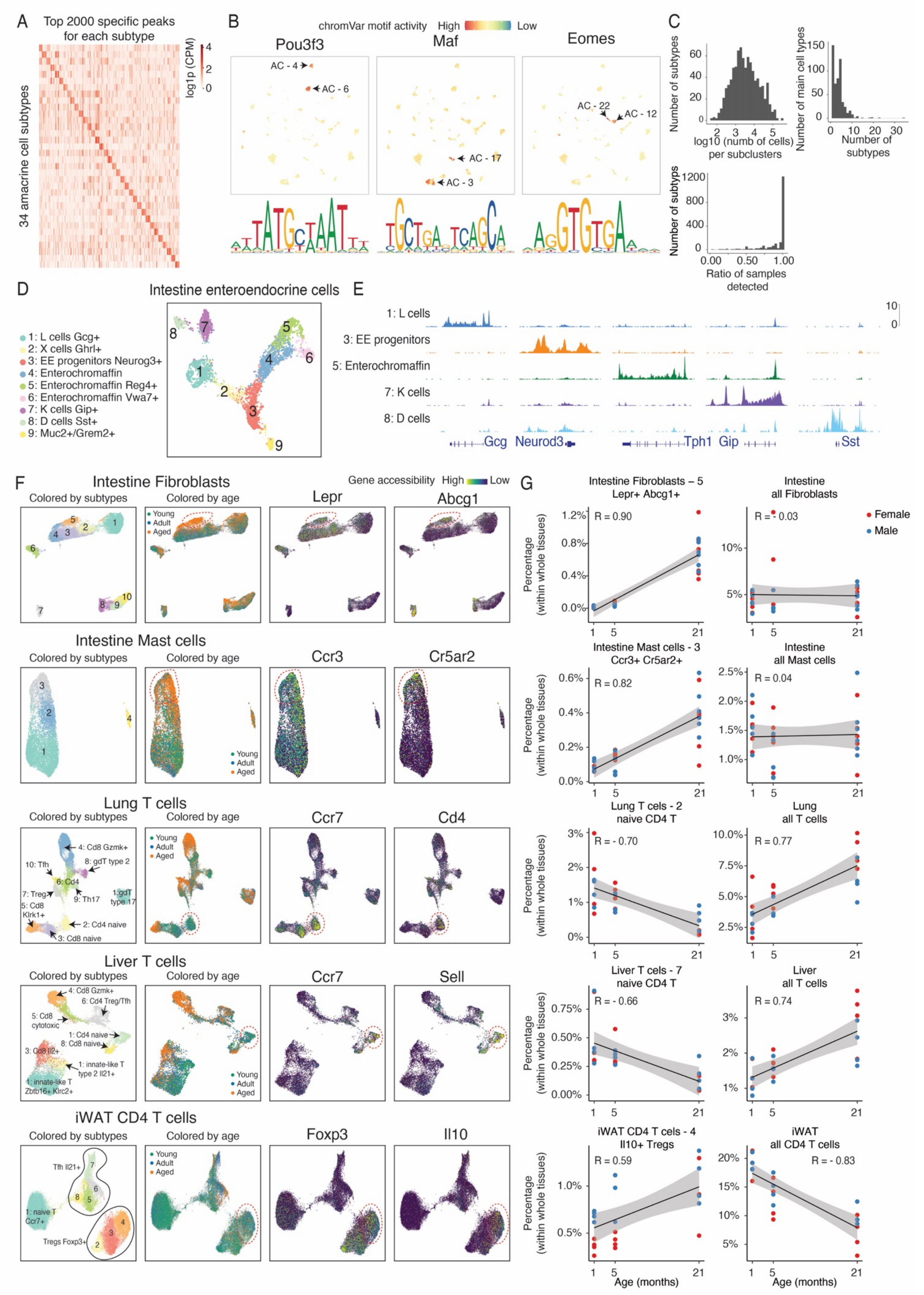

243

bioRxiv preprint doi: https://doi.org/10.1101/2025.05.12.653376; this version posted May 15, 2025. The copyright holder for this preprint (which was not certified by peer review) is the author/funder, who has granted bioRxiv a license to display the preprint in perpetuity. It is made available under aCC-BY 4.0 International license.

Figure S7. Identifications of cell subtypes and associated population changes in aging.
(A) Heatmap showing the aggregated accessibility of peaks specific to each main subtype in amacrine cells, quantified by counts per million.
(B) UMAP visualization of eye amacrine cells, colored by motif accessibilities of example transcription factors specific to distinct subtypes. Motif activities were quantified by chromVar (*21*).
(C) Histograms showing the distribution of cell numbers per subtype (Top left), the number of subtypes per main cell type (Top right), and the fraction of tissue samples that contain cells from each cell subtype (Bottom left).
(D) UMAP visualization of intestine enteroendocrine cells, colored by subtype identity.
(E) Genomic tracks showing the gene accessibilities marking different subtypes of intestine enteroendocrine cells.
(F) UMAP plots showing subclustering results for intestine fibroblasts, intestine mast cells, lung T cells, liver T cells and iWAT CD4 T cells, colored by subtype ID, age group, and accessibilities of genes marking circled subclusters.
(G) Scatterplot showing the proportion changes of indicated subclusters and their parental main cell types with age, along with a linear regression line.

bioRxiv preprint doi: https://doi.org/10.1101/2025.05.12.653376; this version posted May 15, 2025. The copyright holder for this preprint (which was not certified by peer review) is the author/funder, who has granted bioRxiv a license to display the preprint in perpetuity. It is made available under aCC-BY 4.0 International license.

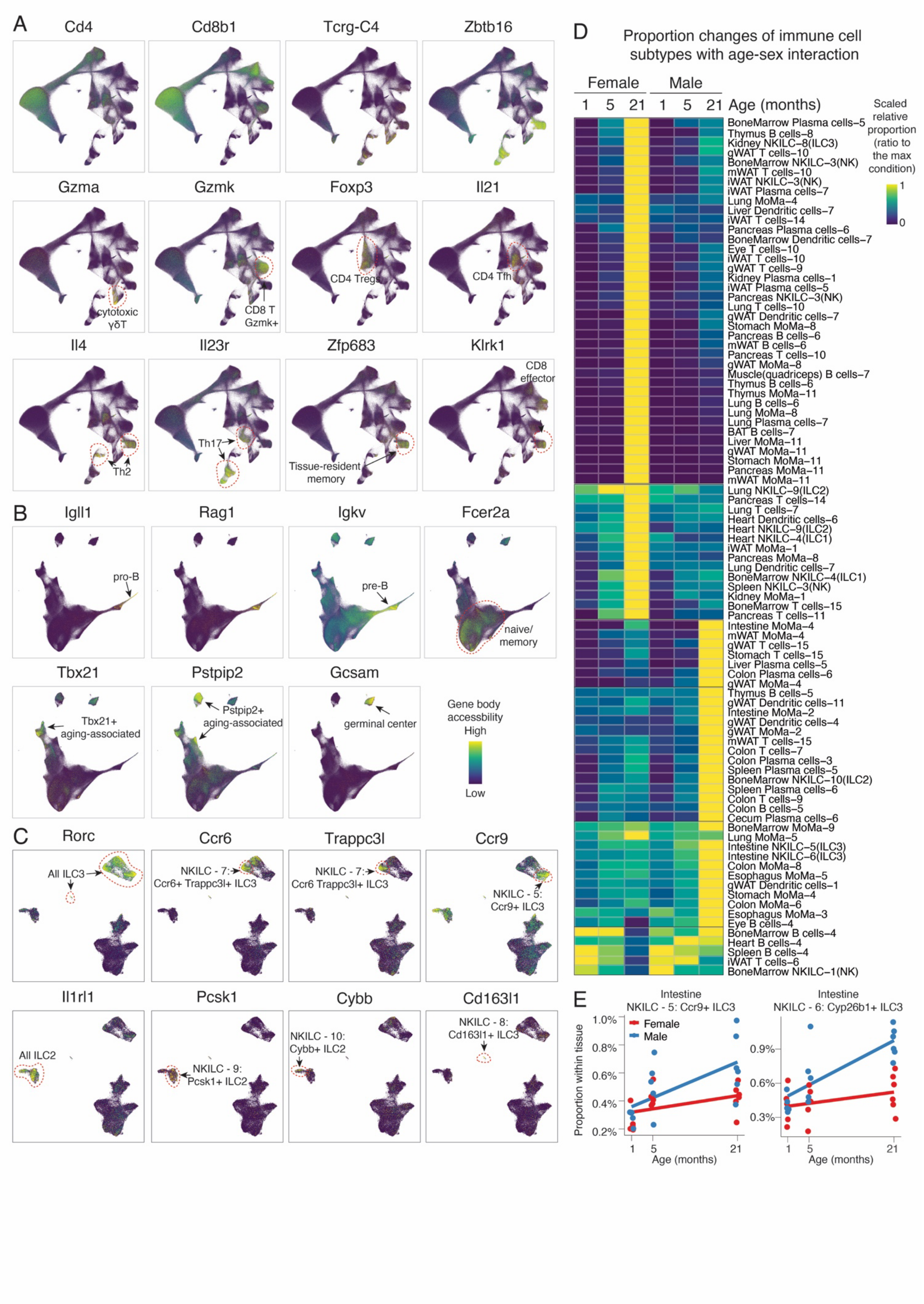

bioRxiv preprint doi: https://doi.org/10.1101/2025.05.12.653376; this version posted May 15, 2025. The copyright holder for this preprint (which was not certified by peer review) is the author/funder, who has granted bioRxiv a license to display the preprint in perpetuity. It is made available under aCC-BY 4.0 International license.

Figure S8. Global characterizations of immune cell states and their proportional changes with age.
(A) UMAP plots showing combined clustering of all T cells across tissues, colored by accessibilities of genes marking distinct subtypes.
(B) UMAP plots showing combined clustering of all B cells across tissues, colored by accessibilities of genes marking distinct subtypes.
(C) UMAP plots showing combined clustering of all innate lymphoid cells across tissues, including NK cells, ILC1, ILC2 and ILC3, colored by accessibilities of genes marking distinct subtypes.
(D) Heatmap of relative proportions of immune cell subtypes with significant age-sex interactions. Rows represent immune cell subtypes within their respective tissues, while columns correspond to mouse conditions grouped by age and sex. For each immune cell subtype within each tissue, its proportion (within each tissue) were first averaged across samples and then normalized to the maximum value within each row.
(E) Scatterplot showing male-baised expansions of *Ccr9*+ ILC3 and *Ccr6*+ *Cyp26b1*+ ILC3 in intestine along aging. Each dot represents one animal, with linear regression lines added for each sex.

bioRxiv preprint doi: https://doi.org/10.1101/2025.05.12.653376; this version posted May 15, 2025. The copyright holder for this preprint (which was not certified by peer review) is the author/funder, who has granted bioRxiv a license to display the preprint in perpetuity. It is made available under aCC-BY 4.0 International license.

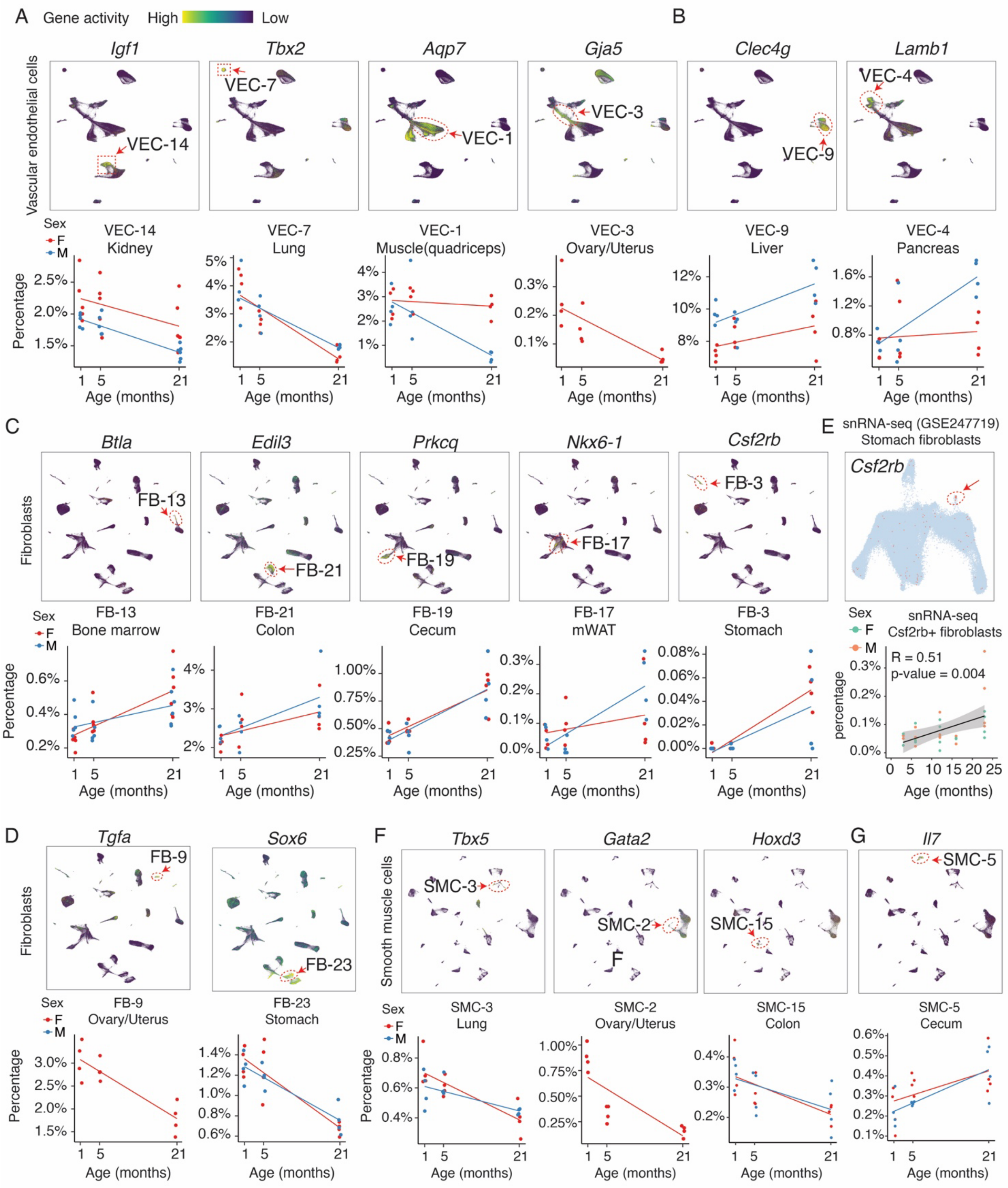

282

bioRxiv preprint doi: https://doi.org/10.1101/2025.05.12.653376; this version posted May 15, 2025. The copyright holder for this preprint (which was not certified by peer review) is the author/funder, who has granted bioRxiv a license to display the preprint in perpetuity. It is made available under aCC-BY 4.0 International license.

Figure S9. Cross-tissue identifications of subtypes for vascular endothelial cells, fibroblasts, smooth muscle cells and their aging-associated population changes.

(A-B) Top: UMAP plots showing the combined clustering results of vascular endothelial cells, colored by the accessibility of genes representing subtypes that expand (A) or deplete (B) with age. Bottom: Scatterplots showing the proportions of the indicated subtypes (normalized to total cells within the tissue) across three age groups, with linear regression lines added for each sex.

(C-D) UMAP plots showing the combined clustering results of fibroblasts, colored by the accessibility of genes representing subtypes that expand (C) or deplete (D) with age. Bottom: Scatterplots showing the proportions of the indicated subtypes (normalized to total cells within the tissue) across three age groups, with linear regression lines added for each sex.

(E) Validation of *Csf2rb*+ fibroblast expansion, as shown in (C), in aged stomach tissue using published snRNA-seq data (*7*). Left: UMAP plot of stomach fibroblasts colored by *Csf2rb* expression. Right: Scatterplot showing age-dependent expansion in *Csf2rb*+ fibroblasts, together with a linear regression line.

(F-G) Top: UMAP plots showing the combined clustering results of smooth muscle cells, colored by the accessibility of genes representing subtypes that deplete (F) or expand (G) with age. Bottom: Scatterplots showing the proportions of the indicated subtypes (normalized to total cells within the tissue) across three age groups, with linear regression lines added for each sex.

bioRxiv preprint doi: https://doi.org/10.1101/2025.05.12.653376; this version posted May 15, 2025. The copyright holder for this preprint (which was not certified by peer review) is the author/funder, who has granted bioRxiv a license to display the preprint in perpetuity. It is made available under aCC-BY 4.0 International license.

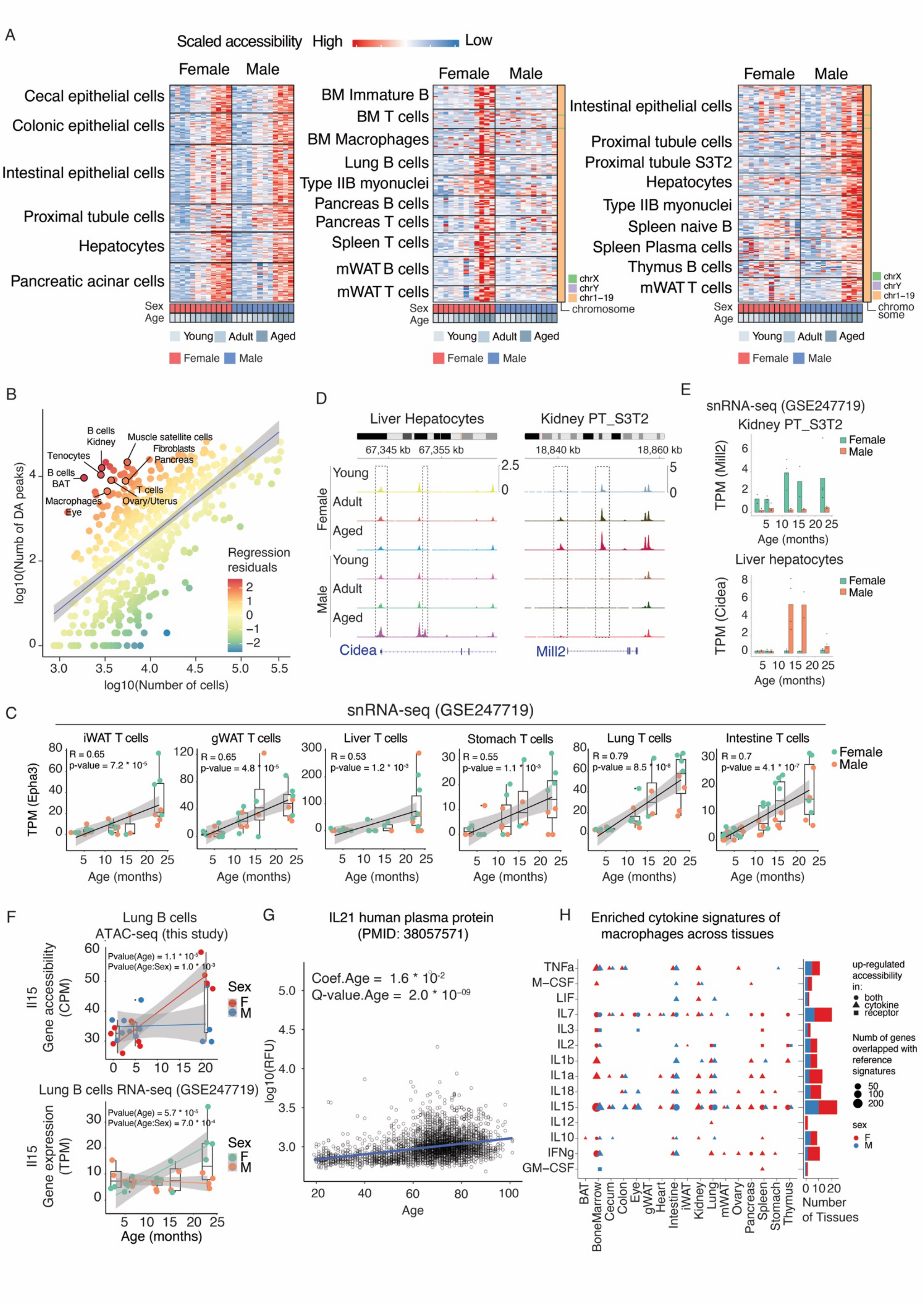

bioRxiv preprint doi: https://doi.org/10.1101/2025.05.12.653376; this version posted May 15, 2025. The copyright holder for this preprint (which was not certified by peer review) is the author/funder, who has granted bioRxiv a license to display the preprint in perpetuity. It is made available under aCC-BY 4.0 International license.

Figure S10. Identifications of cell-type-specific molecular changes in aging.
(A) Heatmaps showing examples of differentially accessible (DA) peaks that are sex-shared (left), female-specific (middle), and male-specific (right). Rows correspond to DA peaks identified in each main cell type, while columns represent individual mice. Peak accessibility was quantified as counts per million for each sample in each cell type.
(B) Scatterplot showing the correlation between the number of cells and the number of DA peaks across cell types, with a linear regression fit. Cell types with high regression residuals are labeled.
(C) Scatterplot showing the increased expression of *Epha3* in T cells across multiple tissues, with a linear regression line, validating the chromatin changes in Figure 6F.
(D) Genomic tracks of male-specific DA peak in liver hepatocytes (left) and female-specific DA peak kidney proximal tubule cells S3T2 (right).
(E) Barplot showing validation of sex-specific chromatin accessibility changes in (D) using gene expression data of the same cell type. Dot represents animals.
(F) Scatterplots showing the increased gene accessibility (top) and expression (bottom) of *Il15* in lung B cells, specifically in females, with linear regression lines added for each sex.
(G) Scatterplot showing the age-related increase in plasma protein levels of IL-21. Data was obtained from (*3*), with a linear regression line included.
(H) Left: Dot plot summarizing cytokine signatures enriched in aged macrophages across tissues. Only signatures supported by accessibility changes in secretion or receptor genes are shown. Right: Bar plot summarizing the number of tissues exhibiting activated signatures for each cytokine, separated by sex.

bioRxiv preprint doi: https://doi.org/10.1101/2025.05.12.653376; this version posted May 15, 2025. The copyright holder for this preprint (which was not certified by peer review) is the author/funder, who has granted bioRxiv a license to display the preprint in perpetuity. It is made available under aCC-BY 4.0 International license.

Supplementary Tables

Table S1. Metadata for animals included in this study.

Table S2. List of gene markers used for cell type annotations.

Table S3.List of cell-type-specific peaks, including the counts per million (CPM) value for the most accessible cell type and the mean CPM across all cell types for each peak

Table S4. Differentially abundant main cell types with age across tissues. “qvalue_age” indicates the significance of a non-zero coefficient for the age term. “qvalue_interaction” indicates the significance of a non-zero coefficient for the age-sex interaction term. “Regression R2” refers to the coefficient of determination of the regression model. “Pearson r” means the Pearson correlation r calculated between cell fraction of each main cell type within their corresponding tissues and ages, including samples of both sexes.

Table S5. Differentially abundant subtypes with age across tissues. “qvalue_age” indicates the significance of a non-zero coefficient for the age term. “qvalue_interaction” indicates the significance of a non-zero coefficient for the age-sex interaction term. “Regression R2” refers to the coefficient of determination of the regression model. “Pearson r” means the Pearson correlation r calculated between cell fraction of each subtype within their corresponding tissues and ages, including samples of both sexes.

Table S6. Differential accessible peaks with age shared in both sexes, including logFC, p-value, and q-value for each peak in both females and males.

Table S7. Differential accessible peaks with age unique to females, logFC, p-value, and q-value for each peak in both females and males.

Table S8. Differential accessible peaks with age unique to males, logFC, p-value, and q-value for each peak in both females and males.

Table S9. Aging-associated linkages with consistent changes in gene expression and chromatin accessibility at promoters and putative cis-regulatory elements. Each row represents a gene-peak-CRE linkage, including the gene name and genomic coordinates of the promoter and linked cis-regulatory site. The column “group” indicates whether the linkage is identified from both sexes or from one sex.
